# Population density as the attractor of business to the place

**DOI:** 10.1038/s41598-024-73341-8

**Published:** 2024-09-27

**Authors:** Katarzyna Kopczewska, Maria Kubara, Mateusz Kopyt

**Affiliations:** Faculty of Economic Sciences, University of Warsaw, Ul. Dluga 44/50, 00-241 Warsaw, Poland

**Keywords:** Economies of density, Attracting mechanism, Population density, Hierarchical agglomeration, Convenience, Business venturing, Statistics, Socioeconomic scenarios

## Abstract

Firm location studies traditionally assume that this is business that attracts other business to a given location. They focus on knowledge transfers between the most productive and innovative firms and analyse agglomeration economies arising from matching, learning and sharing mechanisms. We argue that the population density also attracts firms to the place. Therefore, we extend this stream of research by using the concept of economies of density to discuss the effects of the attraction of firms by population. Our study explores the existence of the hierarchical causal mechanism that occurs when 2nd line firms understood as non-innovative, less productive retail and service businesses together with human settlement create an environment that attracts 1st line firms - the most innovative and productive businesses. We show that population density and business agglomeration jointly affect the location of firms in different sectors and should be considered as complementary rather than substitutable attraction factors.

**Supplementary Information:**

The online version contains supplementary material available at 10.1038/s41598-024-73341-8.

## Introduction

The recent literature on business location has largely focused on the most attractive firms which are knowledge-based, patenting, exporting and growing intensively (we call them 1st line firms), and on the relationships between these firms, including agglomeration externalities, knowledge flows, proximity and spillovers^1,2^. A comprehensive theoretical justification of this micro-world was started 135 years ago by Marshall^3^, developed within New Economic Geography^4,5^ and well-defined mechanisms of matching, learning and sharing^6^. This zoomed in the analytical perspective - even if it explains in detail this most prominent layer of economic activity, the broader context is missing. There are two aspects that need to be addressed: first, the remaining part of firms that are mostly local retail, financial and personal services that provide the economic base and facilities (we call them 2nd line firms, see Appendix 7); and second, human settlement.

Human and business populations constitute the economic landscape of a given place. Even if they do not always participate in matching, learning and sharing mechanisms, their role is to attract others to the place. The concept of the 15-minute city^7^ is to design vibrant urban areas—a mix of residential, entertainment and commercial elements close together, with decentralised “production and consumption hubs”, where densification refers to both population and service provision. In this type of urban structure, people and 2nd line firms create an attractive environment for 1st line firms. It is clear that a high-growth company employing specialised workers will prefer to locate near similar firms^6^. However, given a guaranteed neighbourhood of a similar company, it will prefer an urban area full of amenities to a peripheral or desert location. On the other hand, mono-functional business districts lose their attractiveness^8^, as they offer limited urban facilities without permanent residents. This phenomenon reveals the existence of economies of density, which appear next to economies of agglomeration.

Economies of density can be considered as a type of economies of scale^9^. It is mostly associated with logistics, where typical economies of scale are understood as more routes with the same frequency of use, while economies of density are the opposite, more traffic on the same routes^9–11^. The term is also associated with agriculture where economies of density are economies of scale because neighbouring fields are planted with the same crops^12^. Density is defined in terms of the working population, as the density of work places within the municipality by industries^13^, or the density of development understood as the ratio of floor space to ground area^14^, or as population density being equivalent to demand density^15,16^. Economies of density and size relate area of business activity, density of customers and volume of sales and can be measured differently depending on what is fixed and what is changing^16^. In this paper population density is understood as the number of people settled down in a given place, while economies of density are the benefits to firms from co-locating next to people. This is by analogy with standard definitions of business agglomeration which is a concentration of firms in a given location and agglomeration economies, which are benefits to firms from co-locating next to other firms.

The latest empirical literature on the micro-geography of firms and the attenuation of agglomeration externalities^17,18^ concentrates on local neighbourhoods—surroundings derived individually for each firm and distance-decaying interactions within a narrow radius of a few hundred meters. This stream of research is to be complemented with micro-data on the human population close to firms—to operationalise and understand the population density externalities. We argue that the attracting power of economies of population density enables establishing 2nd line firms that provide daily-life infrastructure, which in consequence, may create an attractive environment for the most innovative 1st line firms.

This paper deals with business location to investigate the existence of population density externalities operating through an attraction mechanism. We hypothesise that there exist two parallel channels of business attraction: the well-known one through business agglomeration externalities and the one described here through population density externalities. They are to operate hierarchically—population density, as the primary cause, attracts all types of firms, the most productive ones (1st line) and those providing the everyday infrastructure (2nd line). In addition, the 2nd line firms serve as a mediator—promoting the emergence of the 1st line firms. This agglomeration hierarchy is not only functional, but also has a spatial dimension, as the elasticity of business agglomeration depends on population density and relative location to cities of different sizes.

We provide empirical evidence for the existence of these mechanisms. We estimate three models: the mediation model, the binary choice model and the elasticity model to demonstrate these phenomena. The mediation model shows causal hierarchical relationships between population, 1st and 2nd line firms. The binary-choice model provides a complete view of the determinants of a business location and is estimated twofold: with probit (linear econometric model) and random forest (non-linear supervised machine learning technique)—which is a novel application in business location research. The elasticity model provides evidence of the strength of the relationships between population density and business agglomeration at the local level of unaggregated point data. The major novelty of the paper is the estimation of models on point data, each with its own individual local density of firms and people.

The remainder of the paper is as follows: Section “Theoretical perspective to attraction mechanism of population density economies” introduces the concept of economies of density as complementary to economies of agglomeration and discusses the impact of population density through an attraction mechanism. Section “Structure of empirical data” presents the data. Section “Methodology of modelling the impact of density and agglomeration externalities on the business location” reviews and proposes methodological approaches to study hierarchical agglomeration, population density externalities and agglomeration elasticity in intra-urban and extra-urban environments. Section “Empirical verification of density-based location decisions model” presents an empirical study of the total population of 1 million (mln) of 1st and 2nd line firms in the NUTS2 Mazovia region (Poland, with Warsaw capital city) including individual point data on the 5.4 million population based on 1 km × 1 km grid data. It shows that population density is an essential element of the business location model. Section “Discussion of the results and conclusions” discusses the implications of population density externalities and presents conclusions.

## Theoretical perspective to attraction mechanism of population density economies

Contrary to the common belief that the agglomeration of firms and the resulting agglomeration economies are the only factors that attract new firms to the area, we show that there are two parallel mechanisms: one, well-known, based on business clusters and agglomeration forces, and second, described here, based on resident population and economies of density. Agglomeration economies, understood as the benefits to firms of co-locating with other firms, have been widely explained by micro-foundation mechanisms of matching, sharing and learning^6^ and studied in detail^19^. These benefits can be understood as a reduction in average costs, financial and technological externalities, increasing returns to scale in production, first-mover advantages, and regional specialisation^20^. On the other hand, we argue that there exist **population density economies**, which are, by analogy, the benefits to firms from co-location to people. To our knowledge, they have not been studied in the literature. As we show, economies of population density can be well explained by the **mechanism of attracting**, which has its roots in the social sciences. This mechanism assumes that high population density attracts 2nd line retail and service firms, creating an attractive urban environment, that consequently attracts the most productive 1st line firms.

This phenomenon can be explained in purely economic terms, as well as in broader, social and spatial terms, all of which are described in more detail below. From an economic perspective, high population density is associated with a large local customer base and a nearby mass of customers. Therefore, a diversity of customer preferences together with a greater chance of satisfying diverse tastes, and low interaction costs in terms of distance and time, activates the attraction mechanism. From a social perspective, which is intertwined with economic issues, it is explained by the higher livability and convenience of a given place. From a spatial perspective, diverse relative locations matter for the strength of the attraction mechanism that generates spatial heterogeneity. These mechanisms are discussed in detail in the following section.

### Economic factors

The urban market has long been perceived as an attractive environment for business. This is mainly due to agglomeration economies associated with external economies of scale (due to co-location)—external to the firm, but internal to the region, and typically divided into economies of localisation (due to the proximity of firms in the same sector) and urbanisation (due to neighbourhood of diverse businesses). These effects are activated by mechanisms of sharing, learning and matching in relation to knowledge and workers. It is usually argued that economies of agglomeration in densely populated areas increase labour and firms’ productivity, stimulate innovation and make consumption cheaper (i.e. by reducing transport/travel costs)^21,22^.

However, a very different set of economic mechanisms come into play when analysing a “desert urban area”, with a population and no settled business. Porter^23^, in his strategy for rebuilding the American inner cities, starts with a precondition for development—a high population density. Its role is to create local demand to be met by 2nd line firms, located nearby, providing the economic base and facilities, and increasing saturation in retail, financial and personal services. This, combined with safety, transport hubs, a strategic location to reach customers efficiently and quickly, and the involvement of the local community in generating economic stimulus, becomes an attracting factor for 1st line firms.

Urban growth based on local consumption is contradicted to urban growth based on exports. Increased local consumption and the development of small retail businesses appear to require high population density. The role of a superior local intra-urban consumption base in attracting high-value businesses and skilled workers^24^ is mentioned along with the three other factors that drive urban growth. These are (i) import substitution with locally produced goods and services, (ii) creating more jobs locally in labour-intensive service sectors, and (iii) creating an attractive environment for local innovation, which can prospectively grow in scope and spatial range. The superiority of the consumption base is understood as the number and variety of available goods and services, including high quality brands, and their convenient availability in time and space. Urban consumption can be seen explicitly in economic terms as shopping and the use of facilities and services, but it can also be considered more broadly.

Glaeser et al.^25^ suggest that ‘demand for city’ and ‘demand for density’ arise when people want to live close together. This is when urban inhabitants find spatial equilibrium through an optimisation process that balances the urban rent premium (city’s impact on housing prices) with the urban productivity premium (city’s impact on business productivity and wages) and the urban amenity premium (city’s impact on life quality). Glaeser’s determinants of the urban productivity premium lie in transport costs and sharing, learning and matching mechanisms, while the urban amenity premium refers directly to the concept of urban liveability, where the convenience of daily life is complemented by access to culture, sports and even the marriage market. All of these urban premiums depend indirectly on population density within the city.

The existence and survival of firms, especially local ones, often depends on the customer base in the neighbourhood—its quality and quantity^26^. In the Hoteling model, the density of stores increases with the density of the population. Empirical studies confirm this mechanism, but add a wealth factor. Wealthier urban districts (with the same population density as poor ones) enjoy higher density and variety of retail and services^27^, as the preferences of richer inhabitants are more heterogeneous^28^. According to Lancaster^29^, the demand for variety can come from two sources: the taste for diversity of individuals, or diversity of tastes of individuals. This is confirmed by empirical studies: denser areas enjoy higher business diversity due to a larger customer base, while more affluent and denser districts have this effect multiplied due to inherited preferences of the rich for diversity. This is consistent with the “consumer city”^25^, further related to “amenity value”^30^ and confirmed by empirical studies^31^.

The dense urban environment should be analysed from two different perspectives: social and business, which are likely to interact. In the social one, this environment consists of local inhabitants, local workers and commuters only passing by. These people can face urban density externalities: positive ones such as variety, convenience, and quick interactions, but also negative ones such as crowding, pollution, etc. Their attitudes towards dense areas result from inherited preferences and utility from urban density externalities and can vary from negative to positive. From a business perspective, “decisions of firms” are taken by people who are the decision-makers in business. The location decisions they make in business may be purely impersonal, but the smaller the firm, the higher the chance that they are related to the personal preferences of the decision-makers and based on the information from the urban environment. The location of the firm then becomes dependent on density, not through a business channel, but through a private channel.

### Convenience

Recent literature appreciates and analyses the convenience of everyday life, often linking it to the concept of a liveable city. Convenience, as an element of life quality, can be defined as the density and diversity of easily accessible urban amenities^32,33^, or broader within the 5D theory of build environment by density, diversity, design, distance to transit and destination accessibility^34^, while the liveable city concept refers to a relationship between people and cities in terms of all people’s living needs^35^.

The concept of the 15-minute city^7^ is to design lively urban areas—a mix of residential, entertainment and commercial elements in close proximity, with decentralised “production and consumption hubs”, where densification refers to both population and service provision. Within this type of urban structure, people and 2nd line firms create an attractive environment for 1st line firms. It is clear that a high-growth company employing specialised workers will prefer to locate near similar companies^6^. However, given a guaranteed neighbourhood of a similar company, it will prefer an urban area full of amenities to a peripheral or desert location. On the other hand, mono-functional business districts are losing their attractiveness^8^, as they offer limited urban amenities without permanent residents. This phenomenon reveals the existence of economies of density, which appear next to economies of agglomeration.

Urban amenities consist of food services (including restaurants, bars, coffee shops, fast foods etc.), convenient shopping (including groceries, bakeries, small shops etc.), financial services (such as ATMs, banks, insurance etc.), leisure and entertainment (as parks, cinemas, theatres, gym, art, etc.), beauty and health services (as hairdressers, doctors, daily spa, pharmacies, etc.), education (as kindergartens, schools, foreign language courses etc.), everyday life services (as laundries, mobile services, cobblers, etc.) and transport services (as bus stops, subways, shared bikes and scooters etc.). They are expected to be available within 5–20 min of walking distance from residential areas and work places to guarantee convenience in a liveable city^36^. The first studies confirm that urban amenities stimulate creativity better than simple firm agglomeration^37^.

Population density is an attracting factor for 2nd line firms, who are responsible for creating urban facilities. Population density accounts for the existence of 2nd line firms, while their diversity increases with people’s proximity. This phenomenon is treated as a basis principle in the urban design literature. Jacobs & Appleyard^38^ underline that urban life requires a minimum mass of people interacting in close proximity. They specify the minimum net density to support urban life as 30–60 people per acre of land, and maximum around 200 persons per acre. However, density without livability may result in slums rather than vibrant cities (see^23^ on inner city problems)(i.e. missing livability in densely populated inner-cities of American cities results often from social problems of inhabitants as high crime rates, low education, low interest in regular work). Therefore a mixture of living, working, shopping, and recreation urban functions must co-exist together, not within a single building, but rather using a variety of public places. The interaction of people that happens in the urban space, mostly through the availability of facilities within walking distance, is considered the main advantage of high-density inner-urban living^39^. Therefore, urban density provokes many studies on quality of life, related to sustainability, liveability, neighbourhood satisfaction etc^40,41^.

Population density through the concept of convenience and a liveable city can explain the location of firms. Regional science theory already decades ago studied convenience. Following Ullmann^42^, “*the problem remains to design cities to take advantage of scale economies and the other advantages of concentration*,* and at the same time to provide optimum liveability*”. Half a century ago, Hägerstrand^43^ also considered the convenience of everyday life. His informal model discusses bundles of daily activities that happen in time and space. He shows that a liveable city, where home, work, facilities etc., are close to each other, allows more activities (bundles) to be performed within the limited time of the day and is the desirable spatial organisation of life, supporting the work-life balance. At the same time, long commuting, which may cause inaccessibility of services (due to distance or opening time), is perceived as an unattractive necessity due to the financial constraints of residents. Even if technological progress and digitalisation of daily life services allow for more flexible management of activities, still many activities need in-person presence. Hägerstrand^43^ implicitly apprises the high agglomeration of 2nd line firms, which follow the population density. This liveable city is treated as an attractive environment for well-paid workers, which implies that the most attractive firms should locate there. Facilities create neighbourhood quality, which was classified by Hägerstrand^43^ as survival, comfort or satisfaction. This quality affects short-run daily activities, cultural participation or access to health care, but also, generates educational paths for children in the long-term. This creates the logical chain in which population density attracts 2nd line firms, and they consequently attract 1st line firms.

Neoclassical economists are troubled by the concept of convenience because it is not easy to operationalise it and might underpin non-rational decisions. Convenience can be measured mainly from the supply side—by knowing if the service is well- placed, with convenient opening hours, delivered reliably, punctually and comfortably^44^. However, the demand side for convenience is less clear. Even if economists assume daily-life decisions to be a logical reason or information-based decision-making process, they would often appear as a normative-affective decision-making model, drawn on emotions, values, normative beliefs, and habits^45^. A growing body of literature shows that convenience is a prerequisite for choice, even if the final decisions are based on affective-emotional reasons, preferences, lifestyles, flexibility, convenience etc^39,46,47^.

### Spatial organisation of regions and cities

Business locates not only in the centres of crowded cities but also in peripheral, sparsely populated areas. Understanding the spatial organisation of regional coverage with population and firms is of high interest. Business-inhabitants interactions started by von Thünen^48^ for regional organisation and Alonso^49^ for the urban organisation have been further developed e.g. to understand the role of agglomeration economies in cities of different sizes^50–52^, to verify the concept of ‘borrowed size’ of offering functions of large cities by smaller second rank cities by networking^50,51^ or to study spatial distributions of population and employment^53^ to understand monocentric vs. polycentric urban design. City size became an important factor in explaining the elasticity of socio-economic phenomena as wages, innovation, rents, amenities, public services, health, wellbeing etc^54,55^. Most of these studies are between cities^56^, do not consider the extra-urban regional scale or do not consider location as an important factor^57^ or operate on urban aggregates^58,59^. Local within-city analyses are rather missing, probably due to the low availability of individual data. Most comprehensive study on elasticities due to density^54^ does not explain the relation between population density and business agglomeration, which makes it a research gap to be filled, especially at the local (not city-aggregated) level. In terms of methodological approach, the problem of business agglomeration elasticity in relation to population density can be compared to the elasticity of wages in relation to business density^56^ or the elasticity of productivity^57^—these studies confirm the significant local agglomeration elasticity, around 0.066 for productivity and 0.03 for wages.

Therefore, two important questions arise: (a) how business agglomeration is related to population density and if the attraction mechanism is working, (b) is this elasticity varying over space, especially due to the relative location (e.g. distance to core, regional or small cities). This is crucial for understanding the spatial hierarchy of agglomeration and density externalities at the micro level, jointly in the intra-urban micro-scale and intra-regional (extra-urban) macro-scale. Elasticity ε is to reveal the fixed parity of business-inhabitants over the territory. One can expect there is local variation in (global) elasticity, revealing the localised externalities. Elasticity ε < 1 prevents extreme overcrowding of land units as doubling the population density would cause a less-than-proportional increase in business units. Elasticity ε > 1 indicates a high attractiveness of the location, higher for firms than for inhabitants. An important question is how the regional diversity of population density translates into the hot and cold spots of business agglomeration.

Economies of agglomeration and density often observed in urban areas, are important drivers of venturing location decisions in entrepreneurial ecosystems and can be studied in terms of their efficiency^60^. Even if regional economists see them as belonging to the individual firm, urban planners already link them with the city and divide the efficiency of agglomeration economies into comprehensive and technical ones^61^. Comprehensive efficiency measures whether a city operates at optimal production efficiency with respect to input factors such as land, labour, and capital. It can be increased by a larger market size, sharing labour pool and infrastructure, shorter travel time and saving resources. Technical efficiency compares cities by scale and can be improved via knowledge spill-overs channels and supply-chain integration. Population density plays a special role there. High population density is a positive factor in increasing market size, saving resources, sharing the labour pool and knowledge spill-overs. However, it can also be a negative factor by increasing congestion and crime, which decrease agglomeration economies’ efficiency. The population density in this setting is one of the fundamental factors, along with land development intensity and road density. All three together matter for city compactness. Large compact cities with high population density provide high urban economic efficiency^61^. Immediate interaction between inputs and outputs follows Jacob’s^62^ logic that local retail and services not only support other businesses, but are also critical to the vitality of community life^63^.

## Structure of empirical data

The empirical verification of the research problem as designed in the previous sections requires a very detailed and extended dataset on the locations of individual firms. There are few general features of the proper dataset: (a) big data, possibly including all registered firms, to obtain reliable estimates and avoid approximations; (b) data from diversified territory, possibly including metropolitan, rural, mid-size and small city areas to capture diverse characteristics and avoid omitted economic environment; (c) data for no-border territory to assure free flow of enterprises and possibly homogenous institutional rules and to avoid natural or institutional location limitations; (d) matching data for population from the same period and at the same granulation level to complement agglomeration with density.

Such conditions were found in the Mazovian region of Poland, which is the central NTS2 region with Warsaw, the capital city. Mazovia has a population of about 5.4 million people and covers a territory of 35,558 km^2^. It has an average population density of 150 persons/km^2^ (varying from 0 to 21,500 persons/km^2^) and a business density of 28 firms/km^2^ (varying from 0 to 16,800 firms/km^2^). The region is interesting because it is similar to small countries, but much less studied in detail. In terms of area, it is slightly larger than countries such as Albania (28,748 km^2^), Belgium (30,528 km^2^), Moldova (33,851 km^2^) and slightly smaller than Switzerland (41,277 km^2^), the Netherlands (42,525 km^2^) or Denmark (43,094 km^2^). In terms of population, it is similar in size to countries such as Ireland (5.27 million), Slovakia (5.43 million), Norway (5.55 million) or Finland (5.6 million). In official EUROSTAT statistics, until 2020 it was a single NUTS2 region and in 2021 it was divided into two statistical NUTS2 regions: Mazovia (Mazowieckie) and Warsaw (warszawski stołeczny). This split resulted from huge income disparities: in 2012 the metropolitan region’s GDP per capita as % of EU27 was 146%, while in the ‘peripheral’ part it was 57%. The population of the region in the last three decades (since the mid’90s) was rather stable, growing ca. 0.3% annually, while the number of firms grew dynamically, by about 4% per year. This makes the region an interesting case for the analysis of business attraction factors.

**Fig. 1:**
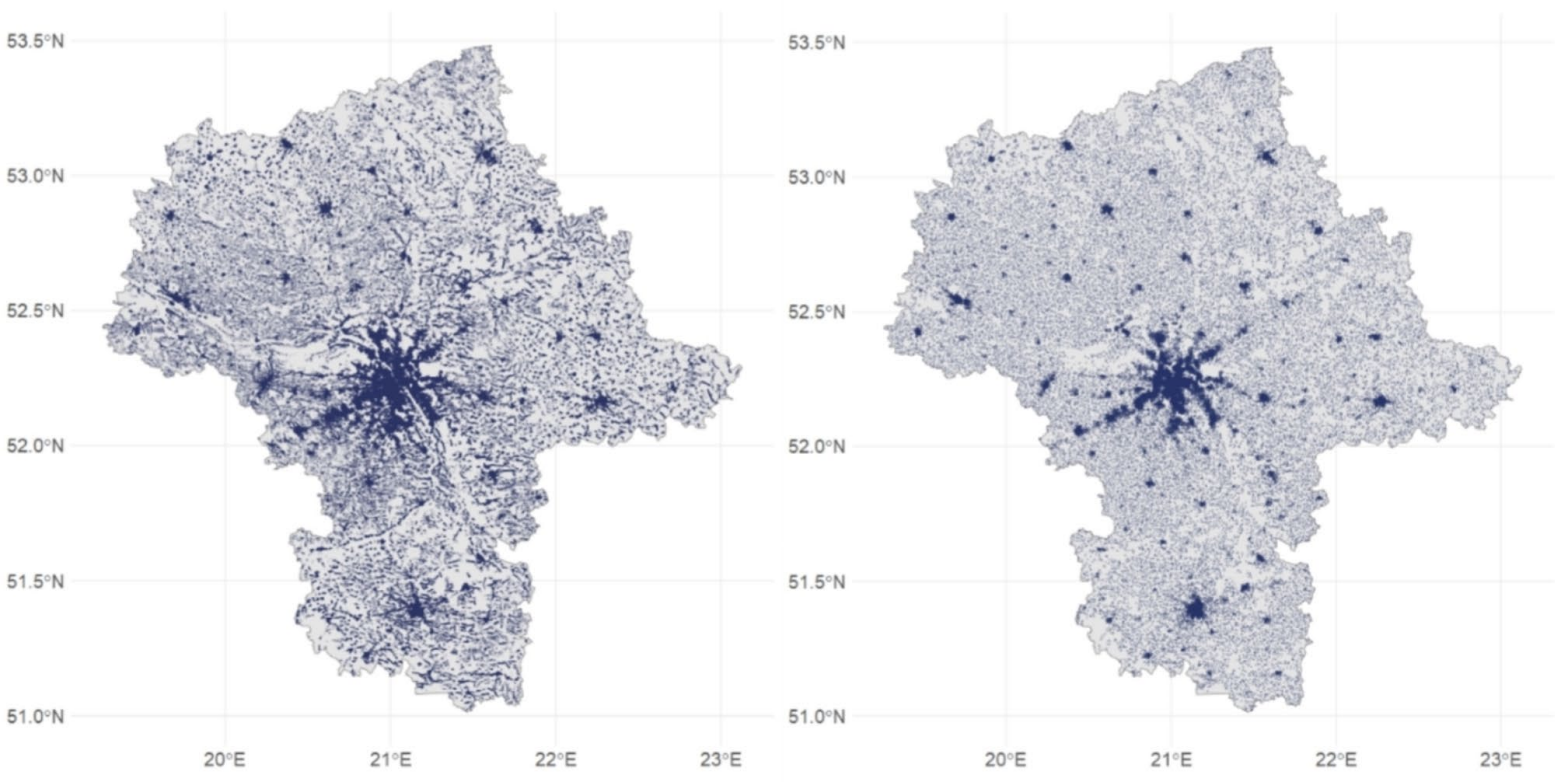
Spatial data used in analysis: (**a**) business location, (**b**) population location.

This study uses full sample (statistical population) data for businesses and inhabitants. The business data include almost 1 million geolocated firms for the Warsaw region in Poland (Fig. 1a), which covers all firms located in this region. Data for 2012 were retrieved from the official register of Polish business entities (REGON) and include address, main sector (indicated with A: U letter and 5-digit code) and employment (in groups 1:5) (Appendix 1). Data were geocoded from postal addresses to latitude / longitude. Supplementary information on the surroundings and features of those businesses are presented in Table 1. Population data (Fig. 1b) were obtained from the official census published in 2012 in a 1 km × 1 km grid with information on the number of inhabitants, age and gender structure in each grid cell (Statistics Poland, 2012).

**Fig. 2:**
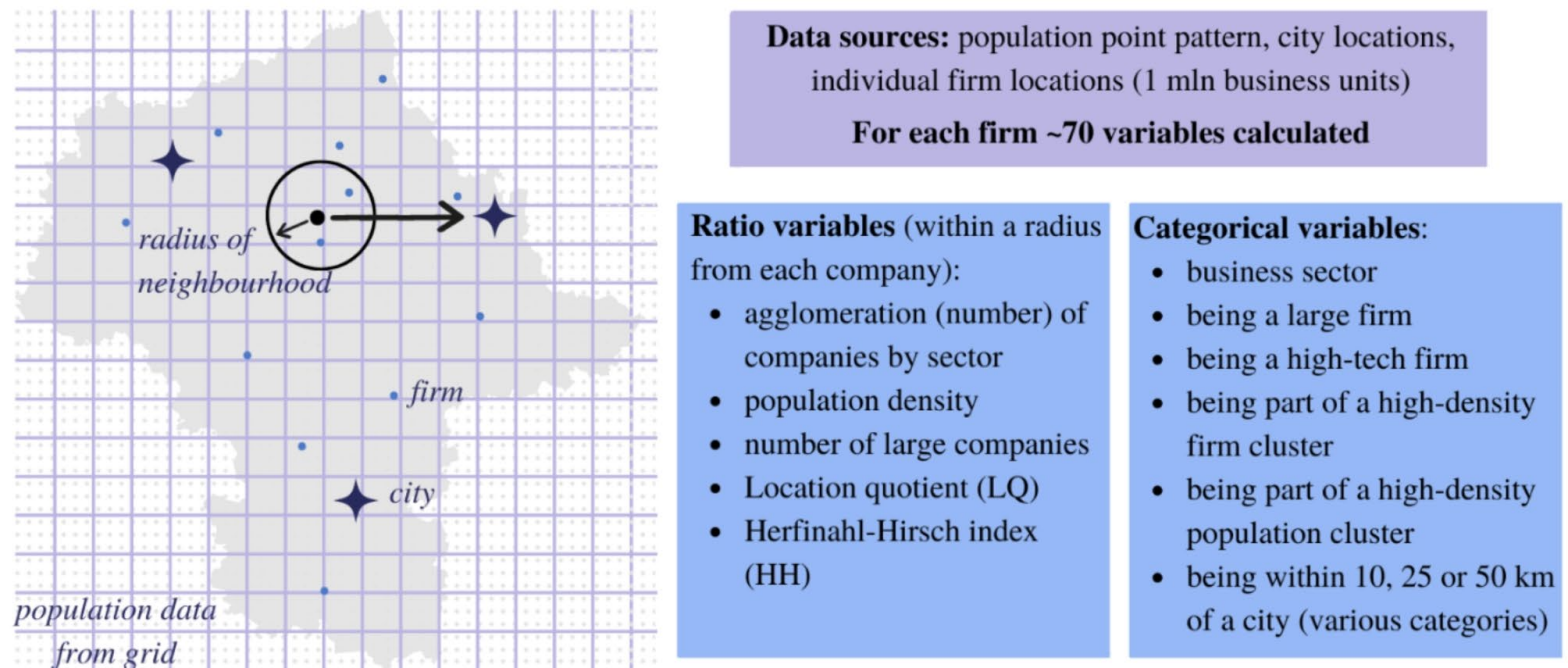
Spatial dimensions of data.

Data on individual firms provide the greatest analytical flexibility. The explanatory power of spatial studies lies in the usa of neighbourhood information. For point data, the most convenient processing method is aggregation within a ring with a radius of a few hundred meters^17^. In this study, we computed for each point out of ca. 1 mln data an individual ring of 500 m and counted what is located there. This is in fact a local individual density for each firm. The local neighbourhoods (denoted as *loc*, see Table 1) were derived for a number of firms by sectors (details Appendix 1) and local indices (described in the next section). This radius is justified by other studies (e.g. for Canada^17^) and allows for a non-zero neighbourhood for a vast majority of points and variables (details in Appendix 3). An alternative radius of 1 km did not change the results. Individualisation of the neighbourhood and lowest spatial granulation known as the micro-geographic approach allows for much deeper insight than typically used data aggregated by regions. Core information on local population density was obtained from census population data available as a grid. The original data, reported as aggregated volumes in 1 km × 1 km cells, were transformed into point data by randomly sampling an equivalent number of points (here 1 point for every 100 persons) within each 1 km^2^ grid box. The location error at 1 km^2^ resolution ranges from a few up to a few hundred meters (ca. 10 minutes’ walk), which remains insignificant for statistical analysis of larger areas (Appendix 3). Population point data were aggregated within the 500 m rings for each firm. Finally, the dataset includes distances from each firm to one of 39 cities in the region (details in Appendix 2). An important aspect of the study was labelling the firms. The division into 1st line and 2nd line firms was approached multiway. In the widest view, 1st line firms are business units from sectors J (Information and communication), K (Financial and insurance activities) and M (Professional, scientific and technical activities). This general group can be narrowed to high-tech firms or KIBS (Knowledge-Intensive Business Services) only. 2nd line firms are remaining non-1st line firms. Figure 2 presents the concept of spatial data processing, while Table 2 reports the details of the created dataset.

**Table 1:** Variables used in the analysis.

Type	Variable name	Description
Location	*dist_core_10*,* dist_core_25*,* dist_core_50*,* dist_midsize_10*,* dist_midsize_25*,* dist_midsize_50*, *dist_regional_10*,* dist_regional_25*,* dist_regional_50*,* dist_localbig_10*,* dist_localbig_25*,* dist_localbig_50*,* dist_localsmall_10*,* dist_localsmall_25*,* dist_localsmall_50*	Dummy variable for each firm: 1 if a firm is located in a radius (10, 25 and 50 km) from the city centre. We considered 39 cities in 5 population size categories that are located within NUTS1 Mazovian region: core 1mln+ (1 city, Warsaw), midsize 100 K+ (2 cities), regional 50 K+ (4 cities), local big 30 K+ (9 cities), local small 15–30 K (23 cities).
	*COREfirms*,* COREpopul*	The dummy variable defines if a given point belongs to a high-density cluster from DBSCAN (of a radius of 0.03° what is equivalent to ca.3,3 km and minPts = 75 firms / 500 persons).
Neighbourhood	*locAggA*,* locAggB*, …, * locAggU*	A number of firms from a given sector (A: U, Appendix 1) calculated in a radius of 500 m from a given firm
	*locAggAgri*,* locAggProd*,* locAggConstr*,* locAggServ*,	Aggregated local sectoral agglomeration values: Agriculture: sector A; Production: sectors B, C, D, E; Construction: sector F; Service: sectors G, H, I, J, K, L, M, N, O, P, Q, R, S, T, U
	*locAggTotal*	A total number of firms calculated in the direct neighbourhood of 500 m of a given firm
	*locHH*	Herfindahl-Hirsh index calculated in 500 m radius
	*locLQ*	Location Quotient calculated in 500 m radius
	*locBIG*	The number of firms classified as big in the direct neighbourhood of 500 m of a given firm
	*locPdens*	A number of inhabitants in the direct neighbourhood of 500 m of the given firm
Features	*Address*,* latitude and longitude*	Postal address of the firm from the REGON register, recoded into geo-coordinates
	*SEC_PKD7*,* PKD7*	Sectoral classification of firm’s main activity from the REGON register
	*empl*,* gr_empl*	Employment size. *Gr_empl* reports five employment classes: up to 9 persons (gr.1), 10–49 persons (gr.2), 50–249 persons (gr.3), 250–1000 persons (gr.4) and 1000 + persons (gr.5). *Empl* gives the approximate mid-value of groups, respectively 5,30, 150, 600 and 1500
	*dummy_if_highetch*	Dummy variable if a firm can be classified as high-tech business
	*dummy_if_big*	Dummy variable if a firm can be classified as big (employment above 250 persons, groups 4 and 5)
	*dummy_agri*,* dummy_prod*,* dummy_constr*,* dummy_serv*	Dummy variable if a firm belongs to one of four main sectors (see details for locAggAgri, locAggProd, locAggConstr, locAggServ)
	1st line firms	Firms from J, K and M sectors
	2nd line firms	Firms from all sectors except J, K and M
	KIBS firms	Firms from sectors K (sections 64–66) and M (sections 70–74)

For estimation, non-dummy variables were standardised. More details in Appendix 1 and 2.

Source: Own work.

The statistical overview of the data (Appendix 3) provides some general observations. Almost all firms are located max. 50 km from a small city, and every third firm not further than 10 km from the core city (33%). Firms prefer locations next to regional cities (50–100 K) than mid-size cities (100 K+). 1st line firms and big firms have denser surroundings than 2nd line firms and mostly are located in high-density clusters of population. 1st line firms in 57% are located in a core city (*r* = 10 km) and 27% in its fringe (*r* = 25 km), while the remaining 16% are in other locations without a clear pattern. 1st line firms differ from 2nd line firms (by sectors) on most variables.

## Methodology of modelling the impact of density and agglomeration externalities on the business location

Recent literature has developed some approaches to the model business location^64^, also conditioned by agglomeration externalities. However, none of them addressed the issues raised in this paper: the hierarchy of agglomeration externalities and the non-linearity of relationships. Thus, even though there are many existing solutions, they are not exhaustive and still need to be complemented.

**Fig. 3:**
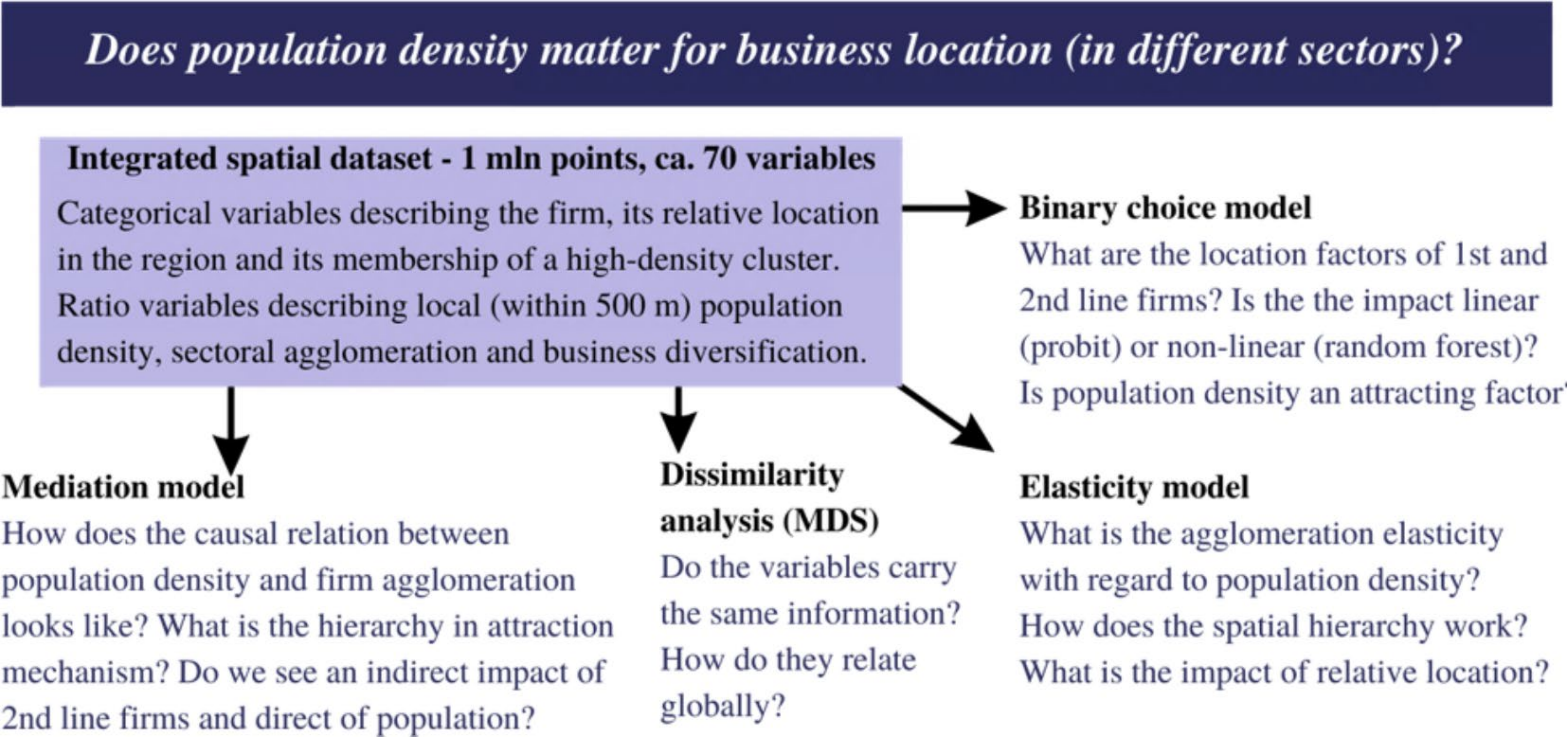
Flow chart of study design. Source: Own work.

We propose three general models: the mediation model, the binary choice model and the elasticity model (Fig. 3). The mediation model, a class of endogeneity tools, aims to detect the hierarchy of impact of population density and business agglomeration on business location decisions. The binary choice model is to detect the determinants of the location of 1st and 2nd line firms. The elasticity model is to assess the elasticity of business agglomeration related to the density of population and localised effects of a relative neighbourhood. All models test the hypothesis that there exist two channels of business attraction: through business agglomeration externalities (matching, sharing, learning mechanisms) and through population density externalities (attracting mechanism). The mediation model by design tests the existence of a causal hierarchical relation of attraction mechanism without including control variables (section “Mediation model to test causality”). Once the causal relationship is proven to exist, binary choice models (probit and random forest) use controls to estimate the impact of density and agglomeration externalities on business location for 1st and 2nd line firms separately (section “Binary choice models: probit and random forest”). The elasticity model is to capture the relation between business agglomeration and population density, controlling for relative location (section “Elasticity model”). These three models explain the existence of 1st and 2nd line firms in a given location—they use factors discussed in section “Explanatory factors”. Estimated equations are discussed in section “General forms of equations for models”. The inclusion of many controls, besides being theoretically justified, requires statistical analysis. Dissimilarity analysis (section “Dissimilarity analysis”) replaces correlation analysis in checking if control variables bring different information, which is to avoid multicollinearity. It is important to underline that all models use as explanatory variables information from the direct neighbourhood aggregated in rings—this is equivalent to classical spatial econometrics using spatial weights matrix W (and models like Spatial Error Model SEM or Spatial Durbin Model SDM). This class of models, micro-geography models, is the appropriate solution for big point data—it avoids the problem of non-scalability of W and non-trivial optimisation of range of neighbourhood^65^.

An important issue is to distinguish between agglomeration externalities and agglomeration phenomenon. Our approach is based on the agglomeration phenomenon (measured as a number of firms), while benefits—agglomeration externalities—stay implicit. This paper does not concentrate on finding what kind of benefits called agglomeration externalities appear for a given firm. However, it takes an implicit and comprehensive effect expressed by location—if firms behave due to the theory where some neighbourhood is profitable, and we observe they locate there, we assume those benefits appear. The same refers to externalities of population density—if a location next to people is profitable and firms locate there, one takes this implicit effect as existing.

The latest empirical literature on micro-geography of firms and agglomeration externalities^17,18^ developed studies concentrating on local neighbourhoods—surroundings derived individually for each firm and distance-decaying interactions within a narrow radius of several hundred meters. This stream of research is to be supplemented with micro-data on the human population close to firms—to operationalise and understand the population density externalities.

### Mediation model to test causality

The problem raised in this paper is the hierarchy of the impact of density and agglomeration externalities on business location. We hypothesise that population density determines the appearance of retail and service less-productive non-innovative 2nd line firms, which in turn attract the most productive and innovative 1st line firms. However, population density itself is also an attractor of 1st line firms. This logic of causality can be tested within the framework of the mediation effect, introduced by Baron & Kenny in 1986 and cited since then ca. 128 K times^66–68^. Using the mediation model we establish a causal connection between population and agglomeration of 2nd line firms and 1st line firms (Fig. 2). The core of the mediation model is a variable called the mediator, which channels the impact of the explanatory variable on the outcome variable. The total impact of the explanatory variable is a total of direct effect (ADE, *Average Direct Effect*) and indirect effect (ACME, *Average Causal Mediation Effect*). This indirect effect is a product of coefficients from two regressions: the first one, where the mediator is explained by the explanatory variable (Eq. 1) and the second one, where the outcome variable is explained by the mediator (Eq. 2):

1 $${mediator}_{i}={\alpha}_{0}+a \cdot {x}_{i}+{\varepsilon}_{i}$$

2 $${y}_{i}={\alpha}_{1}+c\cdot {x}_{i}+b\cdot {mediator}_{i}+{\varepsilon}_{i}$$

which measure the direct effect (coefficient *c*), indirect effect (coefficients *a·b*) and total effect (*a·b + c*). The share of mediated effect is the ratio of indirect and total effects. In this case, the variables are agglomerations in a 500 m radius: *mediator*_*i*_ is the number of 2nd line firms, *y*_*i*_ is the number of 1st line firms, and *x*_*i*_ is the number of inhabitants.

**Fig. 4:**
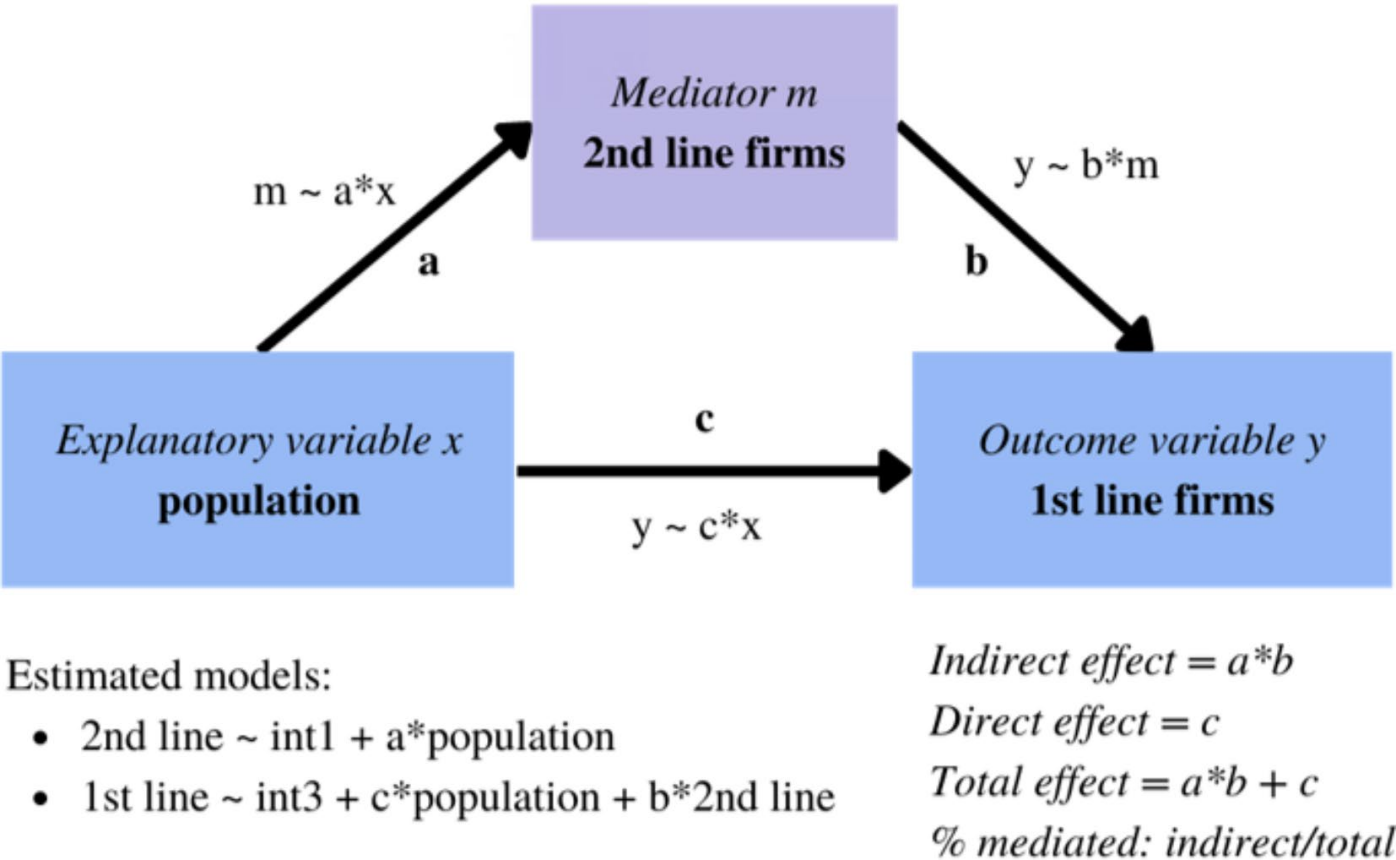
Mediation model. Source: Own work based on Baron and Kenny (1986) (cited ca. 128,000 times).

The motivation for this setting is as follows. High population density creates a large consumer base which becomes a beneficial location for the 2nd line firms (Fig. 4 relation a), what is in line with the liveable city concept. In consequence, entrepreneurial potential stimulated by the increased number of 2nd line companies increases the probability of 1st line firms being opened (Fig. 4 relation b). This relation goes one way only, as much higher capabilities and know-how are needed for opening a 1st line firm. Also, the convenience factor makes highly-skilled and well-earning workers locate next to everyday life facilities. Simultaneously, densely populated areas provide a higher talent pool that can serve as an employee base for 1st line companies. In turn, 1st line firms are more likely to co-locate near highly populated places to maximise the matching mechanism between creative individuals and innovative companies (Fig. 4 relation c).

Some may argue however that this representation of the mediation effect is too simplistic and ignores the complex dimension of bi-directional feedback loops between these phenomena: that people attract business, and at the same time, the business attracts people^54^? Potential endogeneity could arise if people settle down due to jobs in the 1st line firms or increased liveability offered by 2nd line firms. However, the emergence of this relation is highly restricted. The population density in the short term is limited by the available accommodations, preventing any potential impact of businesses on population density. Even in the long term, when the housing market could possibly adapt to the increased demand, it is still bounded by the land available for housing investments, which is a highly scarce resource in the urban space. Therefore the only impact that business can have on the population is on its quality rather than quantity—a process widely known as gentrification^27^. The issue of endogeneity in the mediation model was tested with instrumental variables (IV)^67^ (Appendix 6). We found that the number of flats from the census 25 years earlier is a strong instrument (correlated with population and uncorrelated with business as it dates back to the communistic era), and the accompanying IV regression test evidences no endogeneity in this setting. Our endogeneity tests of this relation are a novelty as previously they were claimed to be very difficult^54^.

### Binary choice models: probit and random forest

Our approach to modelling the decision process of business location follows typical literature design: the entrepreneur decides on establishing a specific firm (1st line, 2nd line, in a given sector) first and, secondly, looks for the best location. This sequence of decisions (not opposite that one has a place and decides on what kind of business to run) can be found in classical, behavioural, NEG and evolutionary models and those theories explain differently the factors making a given location the most desired. The location decisions are binary (yes/no) for a given place. The model is to assess how much a given location is attractive for specific firms. Operationally, the binary dependent variable (*If_to_locate_in_xy*) expresses if the firm in a given location (already known) belongs to a given type (also already known). As the entrepreneurs are rational and not limited in the type and location of business activity and socio-economic environment is known and full information can be obtained, the observed data should express the best entrepreneurial decisions. One estimates a separate model for 1st line firms and individual models for 2nd line firms by sectors (agriculture, production, construction, service). Models for 2nd line firms do not consider the 1st line firms. This approach does not flatten the impact hierarchy and allows for wide control of the local neighbourhood.

The binary choice was modelled with a classical econometric estimation (probit) and supervised machine learning (random forest)^69^. Both approaches used the same formal equation:

3 $$dumm{y}_{locating\in xy}=f\left(set.of.dummy.variables,set.of.standarised.variables\right)+\varepsilon$$

Methodological details of both models are in Appendix 4.

### Elasticity model

The elasticity model is to detect the relationship between population density and business agglomeration in a small spatial scale—a radius of 500 m. Additionally, it can identify local spatial deviations from the main trend resulting from a specific location. Elasticity models have been well-developed in the last two decades, however, they are mostly focused on productivity or wages and rarely considered spatial heterogeneity. The general form of the model is as follows:

4 $$log\left(agglomeration\right)=f\left(log\left(density\right),setoflocationdummies\right)$$

where *log(agglomeration)* is the logarithm of a number of firms located in a radius of 500 m from the analysed firm, *log(density)* is the logarithm of inhabitants in a radius of 500 m, while a set of dummy variables for location explains if a point is in the radius of 10, 25–50 km from the centre of cities of different size or if it belongs to a high-density cluster of firms or population. The model is estimated with OLS as the relation *log(y) ~ log(x)* turned out to be linear. Beta coefficient at density (β_density_) is interpreted as elasticity of agglomeration because of density. In general, one should expect an elasticity coefficient < 1, to assure that agglomeration does not grow to infinity, especially in densely populated areas. However, an elasticity coefficient > 1 may appear in non-overcrowded areas highly attractive for business, e.g. suburbs of bigger cities. Dummy variables reflecting relative location can detect spatially localised variations in agglomeration elasticity.

###  Dissimilarity analysis

Instead of a typical correlation analysis, we have conducted Multidimensional Scaling (MDS). There are a few reasons for that: one is that the dataset contains many dummy variables (for which Pearson correlation does not work) and the other is that correlation analysis refers to selected pairs only, neglecting the joint analysis with other variables in the dataset. MDS is an unsupervised machine learning method to reduce dimensions—it can project the dataset with many more dimensions (variables) into two dimensions (*x*,* y*) and plot it. MDS calculates the multidimensional distance between objects (variables) in the original *k*-dimensional space. Secondly, it assumes *k* points in the new 2D projection and calculates distances between those points (more in Appendix 4).

Outcome—a two-dimensional bubble plot—illustrates the relative similarity of variables (*xy* location), and the impact of a selected variable on the whole setting (bubble size). The bigger the bubble the higher the *Stress Per Point* (*spp*) which reflects the difference between the given variable compared to the other variables considered jointly. Variables with low *spp* are similar to each other and fit the model well. Variables with high *spp* carry very different information in relation to all other variables, even if they reduce the overall fit of the model.

### Explanatory factors

Explanatory variables follow the theory of location decisions. There are at least seven groups of variables which should be included in the model specification. These are as follows. Operational details are in Table 1.

1. **Relative peripherality**—location decisions are driven by relative distance to the cities within the region or distance to the city centre within the city. In many research, the most attractive locations are in Central Business District^70^. Typically, models include distance to the centre of a given city. However, in the case of a region with a dense settlement network and many urbanised areas, this approach is not efficient. First, firms located between core and satellite cities benefit from both urban areas, thus classifying them as gravitating towards one or another city might be misleading. Secondly, non-core cities are often homogeneous and for many firms, it does not matter if they locate close to city A or B if their size is similar. Therefore, we use a set of dummies to express if a given firm is within a specific range (10 km, 25 km, 50 km) from cities of different sizes (core 1 mln+, midsize 100 K+, regional 50 K+, local big 30 K+, local small 15–30 K). The model includes therefore 15 variables (e.g. *dist_core_10*)—for 3 different distance radii to 5 types of cities.
  That allows for the modelling of hierarchical relative location and distinguishing between core urban locations, suburban locations around the main city, locations in second-tier cities and locations outside of main cities. The expectation is that some business sectors which run land-based activities (such as agriculture, forestry, and sectors with large storage areas) prefer peripheral locations. On the other hand, knowledge-intensive sectors concentrate in big cities, due to matching, sharing, learning and attracting mechanisms. There are also public services, that must be provided throughout the entire inhabited territory. This suggests that relative location may be significant/important in a diverse way.
2. **Density externalities**—population density is a core of this study and, until now has rarely been used in explaining business location. The assumption is that the higher the population density in the surrounding, the more attracting power for new businesses which use human capital or are based on sales and/or human interactions. This model studies population density in a narrow radius of 500 m from the firm location (*locPdens*). Recent progress in world-wide population data availability on low aggregation levels (like 1 km^2^ grid) allows for high-precision analysis. Some selected studies used population density but mostly aggregated at the municipality level^71–75^—even though, they evidenced its significant and positive impact.
3. **Agglomeration externalities**—business agglomeration is a fundamental variable in analysing firms’ locations. It measures the number of firms in the surrounding, assuming that the more firms located in the neighbourhood, the higher the attractiveness of the location. For consistency, it should be measured in the same radius as population density (500 m). This approach offers the possibility of deep decomposition into sectors (*locAggAgri*,* locAggProd*,* locAggConstr*,* locAggServ*) and within different radii (for attenuation of externalities what is not analysed in this paper)^17^.
4. **Urbanisation externalities**—the literature is not clear on the operationalisation of the concept of urbanisation externalities. There are some studies^76,77^ that equalise this term with Jacobian agglomeration and take it as the number of firms in the surroundings from the other sectors than the analysed one. However, we see such a definition as closer to local specialisation and diversity, covered in separate points below. In this study, we claim that urbanisation externalities are the effects coming from the high density of population and/or business. This is about the critical mass of people and/or firms required to trigger urbanisation effects. That is why it differs from simple population density or business density and is instead the binary information if a given location belongs to the territory with the highest density or not, referred to a wider surrounding. We treat it as a relative measure by using the DBSCAN clustering algorithm, which indicates locations with the highest density within the analysed territory (Appendix 2). This approach is suitable for point data and simultaneously very flexible and precise, as each point location is classified as a high-density core or as low-density noise. This study considers two variables based on DBSCAN classification: one detecting if an observation belongs to a population high-density cluster (*COREpopul*), and the second for the observation belonging to a business high-density cluster (*COREfirms*).
5. **Local industrial specialisation**—a local neighbourhood of a firm should be assessed not only as mass but also through the structure. The composition of business surroundings has appeared in the literature for a long time^76,77^, and Location Quotient (LQ) is often used as a measure of local industrial specialisation^78,79^. It relates two proportions of business in a given sector compared with all establishments, one in the narrow surrounding and the second over the whole territory. Formally, it is expressed as:
  5 $${LQ}_{i}=\frac{\frac{\sum {N}_{S,radius}}{\sum {N}_{radius}}}{\frac{\sum {N}_{S,region}}{\sum {N}_{region}}}$$
  where subscript *S* stands for the sector, *radius* for the local neighbourhood, and *region* for the whole territory, and *N* is the number of firms. LQ can be understood as the ratio of self-sector shares—in the analysed radius and in the whole dataset. Values of *LQ ≈ 1* suggest that local sectoral composition is similar to general, and no local specialisation is observed. Values of *LQ > 1* (practically *LQ > 1.15*) indicate a concentration of similar businesses in the surrounding, while opposite values of *LQ < 1* (practically *LQ < 0.85*) suggest sectoral loneliness of the firm. In this model, we include LQs for the sector of the analysed firm in a 500 m radius (*locLQ*).
6. **Local industrial diversity**—the local composition of a business neighbourhood can be approached with the Herfindahl-Hirsch index (HH) within a specified radius from the given firm^79^. HH index measures the degree of industrial monopolisation, or oppositely, competition. HH index is expressed as:
  6 $$HH={\sum }_{radius}{s}_{k}^{2}$$
  where *s*_*k*_ is the share of the firm (by sales, employment etc.) in a given sector across the whole territory. This measure calculates the sum of the overall shares for local neighbourhoods for a given sector (as observation). Interpretation of HH values is in commonly accepted thresholds: *0 < HH < 0.15* for high competition and no concentration (high diversity), *0.15 < HH < 0.25* means moderate concentration as there exist from 4 up to 7 firms on the market with equal shares (moderate diversity), while values *0.25 < HH < 1* suggest high concentration or even monopoly (low diversity). In this model, we include HHs for the sector of the analysed firm (*locHH*).
7. **Local competition**—business location decisions may be driven by a willingness to locate next to important business players. These are usually large and well-established companies, employing hundreds of workers and possibly maintaining a high market share. As evidenced by Porter^80^, co-location with big firms may be profitable. This concept can be measured with the agglomeration of big firms in a local neighbourhood of each firm, here in a radius of 500 m (*locBIG).*

### General forms of equations for models

Each of three models requires a separate specification to capture the effects of interest.

**Mediation model**, a form of a structural equation (Eqs. 1, 2), aims to detect a hierarchy in density and agglomeration:

7 $${locAgg2nd}_{i}={\alpha }_{0}+a\cdot {locPdens}_{i}+{\varepsilon }_{i}$$

8 $${locAgg1st}_{i}={\alpha }_{1}+c\cdot {locPdens}_{i}+b\cdot {locAgg2nd}_{i}+{\varepsilon }_{i}$$

where for each observation (firm) the following are measured within a 500 m radius: the number of 1st line firms (*locAgg1st*), the number of 2nd line firms (*locAgg2nd*) and a number of inhabitants (*locPdens*).

**Probit and random forest models** (Eq. 3), applied to explain location decisions, use a dummy as a dependent variable and factors described above as explanatory variables:

9 $$\begin{array}{*{20}c} \begin{gathered} If\_to\_locate\_in\_xy \, \sim \hfill \\ dist \, \left( {core, \, \;midsize,\; \, regional, \, \;localbig, \, \;localsmall} \right){ + } \hfill \\ + \, locPdens + \, \hfill \\ { + }loc\_agglom \, \;\left( {agr,\; \, prod, \, \;constr,\; \, serv} \right) + \hfill \\ { + }COREpopul \, + \, COREfirms + \hfill \\ + \, locLQ + \, \hfill \\ + \, locHH + \, \hfill \\ + \, locBIG \hfill \\ \end{gathered} & \begin{gathered} Decision\; \, if\; \, to\; \, locate\; \, in\; \, given \, \;place \hfill \\ \left( 1 \right) \, \;relative\; \, peripherality \hfill \\ \left( 2 \right) \, \;density \, \;externalities \hfill \\ \left( 3 \right) \, \;agglomeration \, \;externalities \hfill \\ \left( 4 \right)\; \, urbanisation \, \;externalities \hfill \\ \left( 5 \right) \, \;local\; \, industrial \, \;specialisation \hfill \\ \left( 6 \right)\; \, local \, \;industrial\; \, diversity \hfill \\ \left( 7 \right)\; \, local \, \;competition \hfill \\ \end{gathered} \\ \end{array}$$

The detailed form of the equation is given as:

10 $$\begin{aligned} dumm{y}_{firm}&={\beta }_{0}+{\beta }_{1}\cdot {dist}_{core10,i}+{\beta }_{2}\cdot {dist}_{midsize10,i}+{\beta }_{3}\cdot {dist}_{regional10,i}+{\beta }_{4}\\ &\quad \cdot {dist}_{localbig10,i}+{\beta }_{5}\cdot {dist}_{localsmall10,i}+{\beta }_{6}\cdot {dist}_{core25,i}+{\beta }_{7}\\ &\quad \cdot {dist}_{midsize25,i}+{\beta }_{8}\cdot {dist}_{regional25,i}+{\beta }_{9}\cdot {dist}_{localbig25,i}+{\beta }_{10}\\ &\quad \cdot {dist}_{localsmall25,i}+{\beta }_{11}\cdot {dist}_{core50,i}+{\beta }_{12}\cdot {dist}_{midsize50,i}+{\beta }_{13}\\ &\quad \cdot {dist}_{regional50,i}+{\beta }_{14}\cdot {dist}_{localbig50,i}+{\beta }_{15}\cdot {dist}_{localsmall50,i}+{\beta }_{16}\\ &\quad \cdot {locPdens}_{s}+{\beta }_{17}\cdot {locAgg}_{agri.s}+{\beta }_{18}\cdot {locAgg}_{\prod ,s}+{\beta }_{19}\cdot {locAgg}_{constr,s}\\ &\quad +{\beta }_{20}\cdot {locAgg}_{serv,s}+{\beta }_{21}\cdot {CORE}_{firms}+{\beta }_{22}\cdot {CORE}_{popul}+{\beta }_{23}\cdot {locLQ}_{s}\\ &\quad +{\beta }_{24}\cdot {locHH}_{s}+{\beta }_{25}\cdot {locBIG}_{s}+{\varepsilon }_{i} \end{aligned}$$

where *dummy*_*firm*_ is a binary variable indicating if a given firm belongs to a selected group (y = 1) or not (y = 0)—groups defined as 1st line high-tech firms, 1st line KIBS 2nd line agriculture, 2nd line production, 2nd line construction and 2nd line service. Variables were defined as in section “Explanatory factors”, ending .s is for standardised variables (other variables are dummies), the dataset for 2nd line firms excludes 1st line firms.

**Elasticity model** tests the relation between logarithms of business agglomeration (dependent variable, *log(locAggTotal)*) and population density (explanatory variable, *log(locPdens)*), using additional controls—relative location dummy variables defined in the “relative peripherality” group (Eq. 4). The estimated equation is in the form:

11 $$\begin{aligned} log\left(locAggTotal\right)&={\gamma }_{0}+{\gamma }_{1}\cdot log\left(locPdens\right)++{\beta }_{1}\cdot {dist}_{core10,i}\\ &\quad +{\beta }_{2}\cdot {dist}_{midsize10,i}+{\beta }_{3}\cdot {dist}_{reg10,i}+{\beta }_{4}\cdot {dist}_{locbig10,i}\\ &\quad +{\beta }_{5}\cdot {dist}_{locsmall10,i}+{\beta }_{6}\cdot {dist}_{core25,i}+{\beta }_{7}\cdot {dist}_{midsize25,i}\\ &\quad +{\beta }_{8}\cdot {dist}_{reg25,i}+{\beta }_{9}\cdot {dist}_{locbig25,i}+{\beta }_{10}\cdot {dist}_{locsmall25,i}\\ &\quad +{\beta }_{11}\cdot {dist}_{core50,i}+{\beta }_{12}\cdot {dist}_{midsize50,i}+{\beta }_{13}\cdot {dist}_{reg50,i}\\ &\quad +{\beta }_{14}\cdot {dist}_{locbig50,i} +{\beta }_{15}\cdot {dist}_{locsmall50,i}+{\varepsilon }_{i} \end{aligned}$$

where *log(locPdens)* identifies the elasticity of agglomeration with respect to population, while dummy variables for distance from cities are to detect local instability of elasticity.

## Empirical verification of density-based location decisions model

**Fig. 5:**
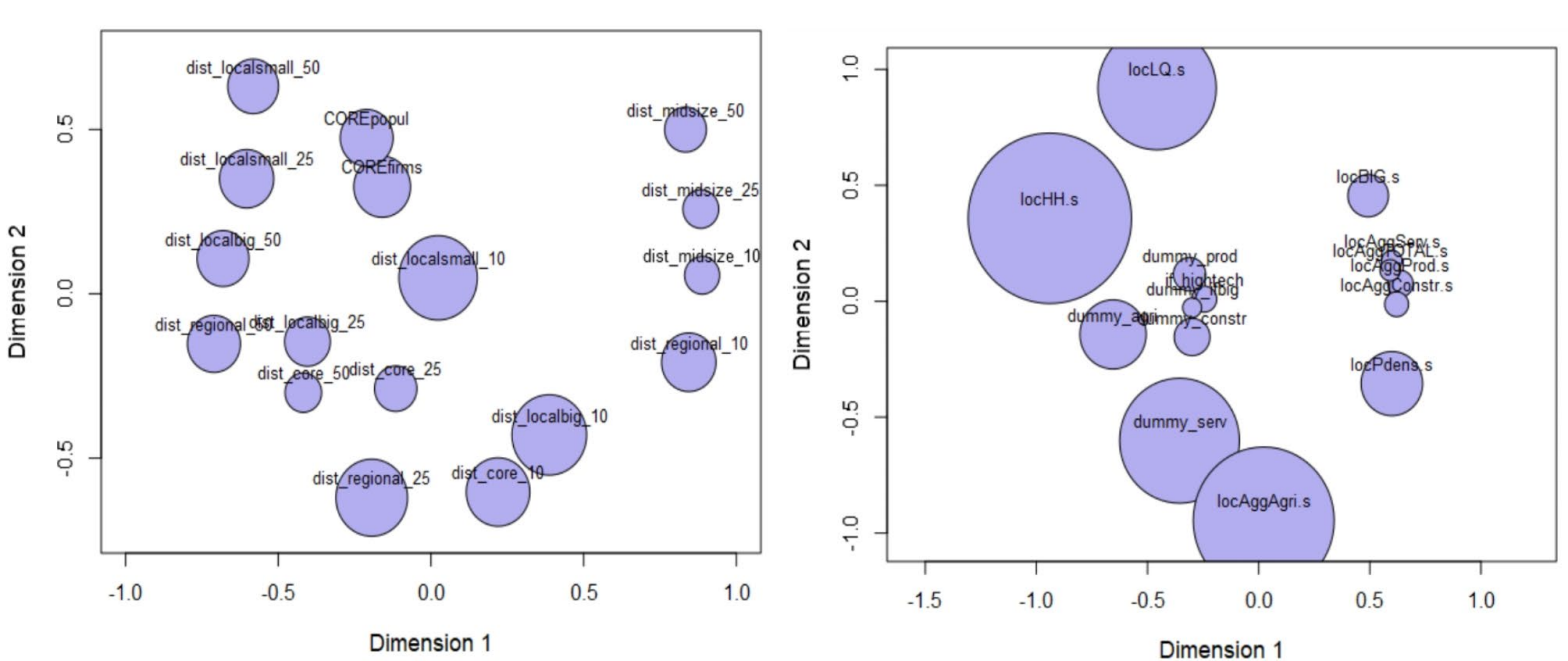
Dissimilarity of variables: (**a**) for distance, (**b**) for neighbourhood. Note: Dimensions 1 and 2 are MDS projections and illustrate proximity of variables (in pairs). The circle’s size (bubble) is the stress-per-point (spp)—the greater the more diverse information included in the variable.

Below we present the empirical results for models and methods presented in section “Methodology of modelling the impact of density and agglomeration externalities on the business location” on data described in section “Structure of empirical data”. **MDS** (*multidimensional scaling*) dissimilarity analysis reveals that among location-related dummy variables (belonging to a high-density area and being within the radius of 10 km, 25 km and 50 km from big, midsize, regional and small cities) each variable carries different information (Fig. 5a)—the bubbles are well separated. Being located within a core city (*dist_core_10*) and being located in the area of a high-density of firms or people (*COREfirms*,* COREpopul*) is not the same information and all of them are very influential (due to the large size of the bubble). In the group of neighbourhood and self-characteristics variables (Fig. 5b) it is visible that population density in the surrounding of firms (*locPdens*) matters a lot and does not overlap with a density of sectoral agglomeration (*locAggAgri*,* locAggProd*,* locAggConstr*,* locAggServ*) or surrounding of big firms (*locBIG*). MDS analysis clearly shows that the inclusion of population density is an important factor in understanding business location and that all the variables taken into account bring new diverse information.

**Fig. 6:**
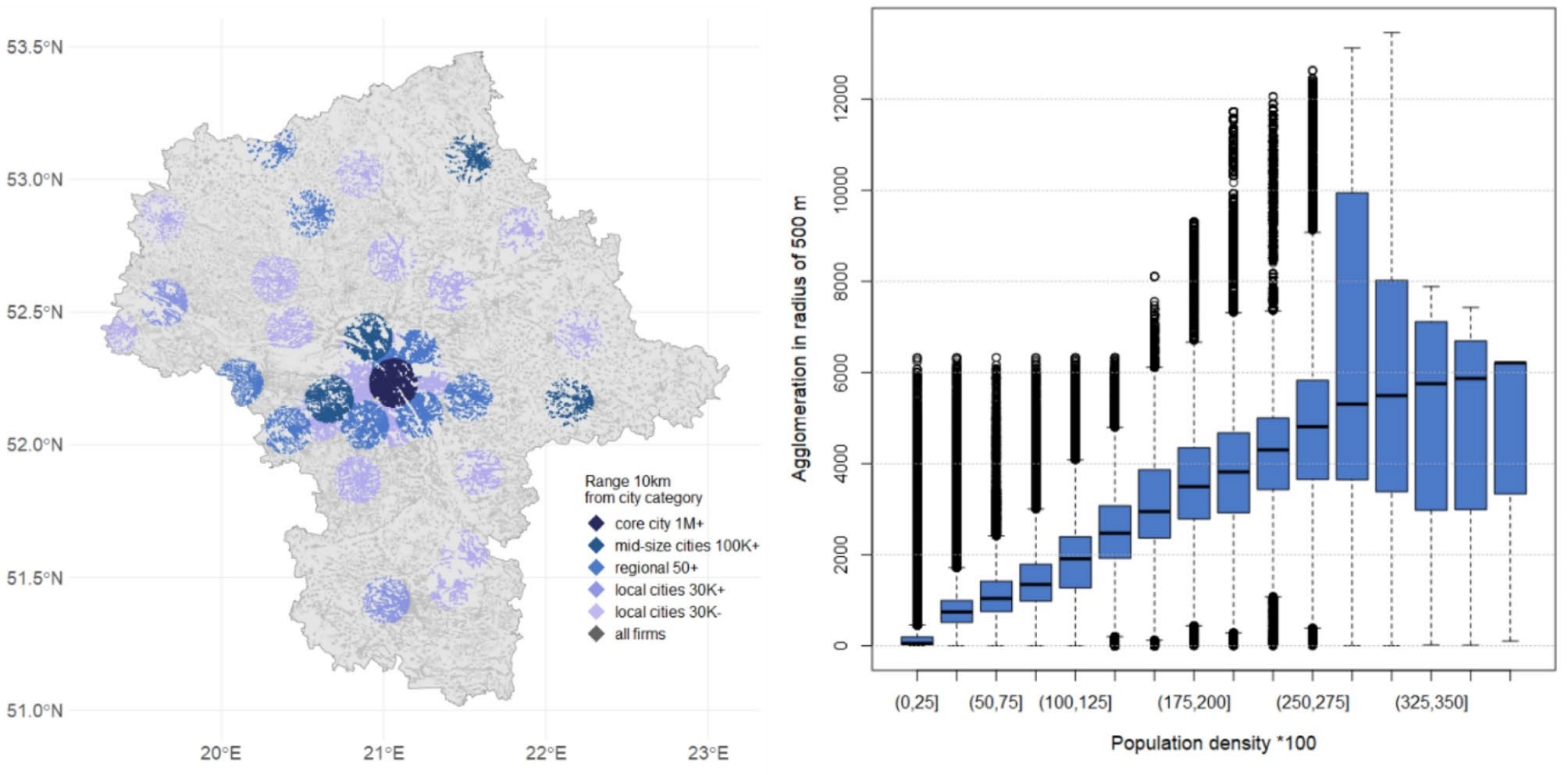
Relative spatial distribution of firms: (**a**) to city centres, (**b**) to population density.

**Statistical inspection** shows that around 70% of firms are located within 10 km from the city centre (Fig. 6a), while the remaining 30% is much more peripheral. There exist a clear relationship between population density and business agglomeration (Fig. 6), which is non-linear and with changing variability (Fig. 6b). Under-trend non-linearity appears at the highest density—this can be justified by the limited capacity of available real estate in locations massively inhabited by people and firms. Increased variance (with density) is also typical for high-density areas—the diversity of highly populated areas in terms of attracted business is much higher than in low-density locations. This suggests the existence of additional factors (e.g. feedback loops, knowledge spillovers, diseconomies of urbanisation, etc.) in high-density areas in attracting business to that place. It also suggests the existence of diverse spatial structures.

**Fig. 7:**
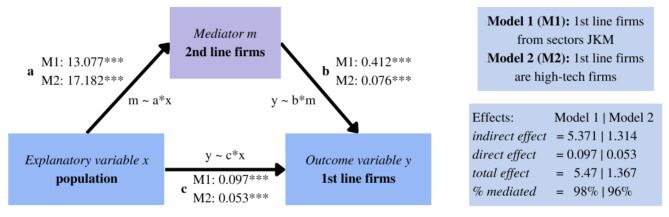
Mediation effects.

**The mediation model** aimed to test if there exists mediation (indirect) and direct effects between business location and inhabiting population. We verified this hypothesis threefold: with two models on point data (Table 2; Fig. 7), and using Poland-wide municipalities data (Appendix 6). All approaches show a significant mediation effect as assumed, justifying the design of further analyses. Moreover, the general model for municipalities was extended to include instrumental variables to eliminate a potential reverse direction reaction (Appendix 6)—the results ensured the validity of the causal relation between population, 1st and 2nd line firms while proving that there is no endogeneity issue in the mediated relation.

Mediation models on point data (Eqs. 7, 8, Table 2) use firm as the unit of analysis and its neighbourhood within a radius of 500 m. The explanatory variable in both models is the population density in firms’ surroundings. In the first model, the mediator variable is the local agglomeration of 2nd line firms (firms from all sectors except J, K and M—details in Appendix 1), while the outcome variable is the local agglomeration of 1st line firms (from J, K and M sectors). In the second model, the mediator variable is the local agglomeration of non-high-tech firms, while the outcome variable is the local agglomeration of high-tech firms (Table 1). In both cases mediation effect and direct effect are significant. The size of the mediation effect was estimated at ca. 96–98%. Results from the mediation model show that the population density is a direct cause of the firm agglomeration, while the effect on the 1st line firm location is mediated by the 2nd line companies creation.

**Table 2:** Mediation effects (visualised in Fig. 7).

	Specification 1	Specification 2
Mechanism	Coefficients from model 1 1st line firms from J, K,M sectors	Coefficients from model 2 1st line firms are high-tech firms
Indirect effect (ACME, *Average Causal Mediation Effect*)	5.371*** = (13.077*** · 0.412***)	1.314*** = (17.182*** · 0.076***)
Direct effect (ADE, *Average Direct Effect)*	0.097***	0.053***
Total effect	5.468***	1.367***
% mediated	98%	96%

Note: Total indirect effects are the product of coefficients (a·b) of two mediator models (m ~ a*x and y ~ b*m); Total effect is the sum of coefficients (a·b + c): total indirect (a·b) and total direct (c) effects; Proportion of mediated effect (%) is the ratio of indirect effect (a·b) to total effect (a·b + c).

**Probit model and random forest** model were estimated using Eqs. 9, 10 (Table 3, Appendix 4, 5, 9). Non-dummy standardised data allow comparison of coefficients between models and interpretation of changes by 1 on a standard deviation scale.

The general conclusion from the **probit model** estimation can be summarised as follows. **Population density** (*locPdens*) matters for the business location of all firms. Its impact is positive (increasing probability) in the case of 1st line high-tech firms and KIBS and 2nd line service sector due to sectoral preferences towards highly urbanised areas. The firms from other sectors (agriculture, construction, production) locate in less dense areas (negative impact, decreasing probability, preferred density is below average). Additionally, being located in high-density cluster also matters a lot—this is measured with dummies from DBSCAN if the location belongs to a high-density core or low-density noise and it applies both to the density of inhabitants (*COREpopul*) and firms (*COREfirms*). 1st line firms, 1st line KIBS and 2nd line production, construction and service prefer high-density locations and mostly high population density matters way more than high-density cluster of firms. This evidences the importance of the attraction mechanism of the population across different business sectors. **Sectoral agglomeration** is significant in the Marshallian way (firms surrounded by the same sector)—2nd line production, construction and service sectors have similar significant coefficients of 0.32–0.34. Jacobian relations understood as a diversified neighbourhood, are also visible—nearby agglomeration from other sectors is significant, but mostly with negative (*loccAggAgri*,* loccAggProd*,* loccAggConstr*,* locAggServ*).

Interestingly, 1st line firms, KIBS and agriculture tend to create high local specialisation (coefficient at *locLQ* > 0), while other firms prefer the local LQ below the average LQ (more diversified environments), as shown by negative coefficients. This suggests strongly Marshallian agglomeration in 1st line firms and agriculture and more Jacobian composition in other sectors. High industrial diversity (*locHH*) appears in 2nd line agriculture and construction due to many big firms. Coefficients are approaching zero for 1st line firms, and 2nd line production and service, which suggests many small firms on the market. There is no clear pattern corresponding to the proximity to big firms. Negative coefficients (*locBIG*) for 1st line and 2nd line service models should be associated with the highest average values in these sectors (Table 3) and a downward correction towards the general average. 1st line firms, KIBS, 2nd line service and production firms tend to locate around cities of any size. For service, there is a clear preference to locate in proximity to bigger settlements (within the radius of 10, 25–50 km around core, midsize or regional cities), while the local cities (below 45 K inhabitants) are much less preferred. The production sector prefers midsize cities (100 K+) within any radius and or local cities as second-best. The agriculture sector in general escapes from any city, tending towards peripheral and rural areas. Coefficients for the construction sector are very weak, what may suggest no strict preferences. Estimation evidence that location attractiveness increases in the surrounding of any type of city, except for agriculture, which deliberately seeks out peripheral areas.

Results from the location choice model support the core concept of this paper, showing how population density matters for different business sectors. Although e.g. service and agriculture sectors have many different preferences towards the proximity of deeply populated areas, population density plays an undeniably important role in any business location. The presented models are based on the best specification, while alternative estimates were presented in Appendix 8. It shows the models without population data—it reveals the bias of remaining coefficients, measured as the difference between right and biased model: (beta_biased/beta_right)-1. The effects of omitted population variables are the strongest at dummies for high density of firms (1.17) and distances to cities (in plus: 2.82, 0.65, 0.53, 0.45; in minus: −0.4, −0.58, −0.25) and visible at sectoral agglomeration variables (−0.94, −0.2, −0.14). It should be noted that the coefficients in the probit models must be interpreted jointly (as conditional marginal changes), and they suffer from partial non-linearity and spatial heterogeneity. The relationships are not the same throughout the territory, as shown by the significance of the dummy variables for the locations. The random forest model ensures the robustness test of the results.

**Table 3:** Estimation results of probit models for 1st and 2nd line firms.

Variable	1st line firms	1st line KIBS	2nd line Agri	2nd line Prod	2nd line Constr	2nd line Service
Intercept	−2.86***	−2.32***	−372.91***	1.91***	−1.71***	−0.79***
Local population density (locPdens.s)	0.02***	0.05***	−0.43***	−0.09***	−0.05***	0.15***
Local agglomeration of agricultural firms (locAggAgri.s)	0	0.01***	0.18***	−0.02***	−0.02***	0.02***
Local agglomeration of production firms (locAggProd.s)	0.01	−0.11***	−0.02	0.34***	−0.05***	0.01
Local agglomeration of construction firms (locAggConstr.s)	−0.10***	−0.13***	0.59***	−0.07***	0.33***	−0.30***
Local agglomeration of service firms (locAggServ.s)	0.11***	0.22***	0.85***	−0.28***	−0.32***	0.32***
Local agglomeration of big firms (locBIG.s)	−0.02***	0.02***	1.20***	0	0.07***	−0.02***
Local Herfindahl-Hirsch index (locHH.s)	−0.07***	−0.006*	−17615.15***	0.03***	−1.56***	−0.01***
Local Location Quotient (locLQ.s)	0.13***	0.04***	0.68***	−0.14***	−0.33***	−0.20***
Dummy for location in high-density cluster of firms (COREfirms)	0.25***	0.28***	−0.37***	0.01	0.02	0.59***
Dummy for location in high-density cluster of people (COREpopul)	0.47***	0.46***	−0.29***	0.21***	0.10***	0.38***
Dummy for location up to 10 km to core city (dist_core_10)	0.06***	0.11***	0.15***	−0.02**	−0.01	0.04***
Dummy for location up to 10 km to midsize city (dist_midsize_10_	0.04	0.06***	0.06***	0.08***	−0.01	0.12***
Dummy for location up to 10 km to regional city (dist_regional_10)	−0.01	0.03***	−0.22***	0.02*	0.01	0.03***
Dummy for location up to 10 km to local bigger city (dist_localbig_10)	−0.11***	−0.07***	−0.06***	0.03***	0.02***	−0.04***
Dummy for location up to 10 km to local small city (dist_localsmall_10)	0.03***	0.05***	−0.01	0.02**	0.03***	−0.04***
Dummy for location between 10 and 25 km from the core city (dist_core_25)	0.19***	0.19***	−0.16***	0.07***	0.01	0.23***
Dummy for location between 10 and 25 km from the midsize city (dist_midsize_25)	0.12***	0.18***	−0.15***	0.13***	0.11***	0.12***
Dummy for location between 10 and 25 km from the regional city (dist_regional_25)	0	−0.02**	−0.07***	0.03***	0	0.09***
Dummy for location between 10 and 25 km from the local big city (dist_localbig_25)	0.16***	0.09***	−0.18***	0.06***	0.06***	0.17***
Dummy for location between 10 and 25 km from the local small city (dist_localsmall_25)	−0.01	−0.01	−0.10***	0.01	0.05***	0.07***
Dummy for location between 25 and 50 km from the core city (dist_core_50)	0.15***	0.13***	−0.23***	0.10***	0.08***	0.11***
Dummy for location between 25 and 50 km from the midsize city (dist_midsize_50)	0	−0.04**	−0.29***	0.06***	0.06***	0.13***
Dummy for location between 25 and 50 km from the regional city (dist_regional_50)	0.07***	0.01	0.10***	−0.06***	−0.09***	−0.04***
Dummy for location between 25 and 50 km from the local big city (dist_localbig_50)	−0.03	−0.01	−0.05***	−0.02**	0.05***	0.07***
Dummy for location between 25 and 50 km from the local small city (dist_localsmall_50)	0.11*	0.06	−0.01	0	−0.07**	−0.09***
AIC	322492.2	578,387	424002.8	412808.3	480026.7	969777.9
McFadden R2	0.076651	0.08353	0.613474	0.027648	0.052229	0.236849
BIC	322,799	578,694	424308.5	413113.9	480332.3	970083.5
Log Likelihood	−161,220	−289167.7	−211,975	−206,378	−239,987	−484,863
Deviance	322440.2	631,053	423950.8	412756.3	479974.7	969725.9
Num. obs.	983,719	983,719	941,378	941,378	941,378	941,378

P-value note: ***<0.001< **<0.01< *<0.05; models estimated using glm() in R as GLM (Generalised Linear Model) framework using a probit link function, with standard errors on the link scale.

**Fig. 8:**
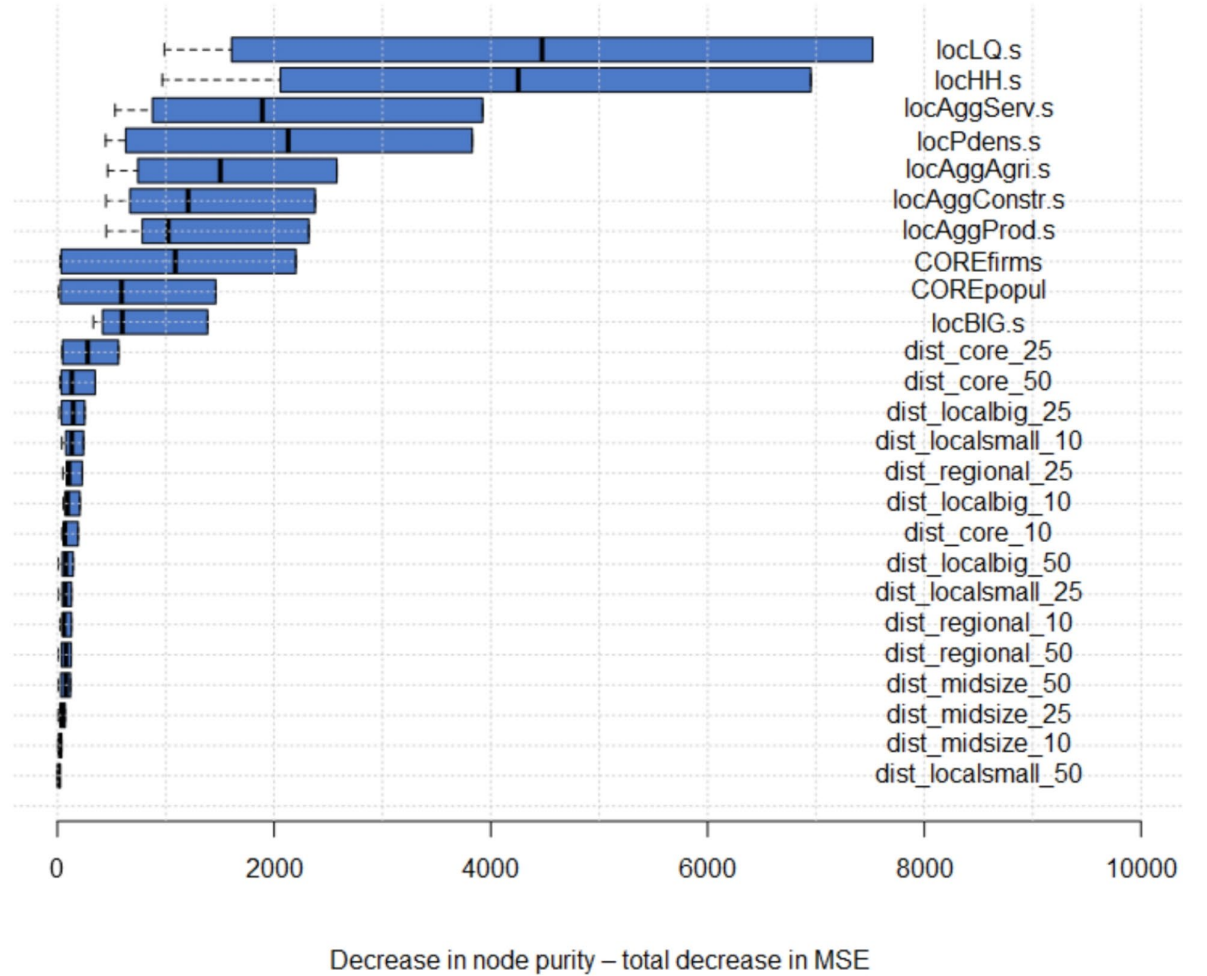
Variable importance from Random Forest model for 1st line, KIBS and 2nd line firms models.

We verified the non-linearity present in spatial relations by estimating a **random forest** model for the same set of equations (Eqs. 9, 10). Supervised machine learning models are presented by the importance of variables (Fig. 8, Appendix 4). Explanatory factors vary between the models, confirming that there is no unique set of location decision factors and that the intrinsic characteristics of business determine which set of location determinants matters. Population density—the core issue of this paper—is relevant in all models. Its importance is highly evident both in population density measured as the number of inhabitants around the firm (*locPdens*) as well as in a dummy if a firm is located in a high-density cluster of firms (*COREfirms*) and population (*COREpopul*). A comparison of probit and random forest models shows that they are highly consistent—indicating that the results are robust and insensitive to estimation methods.

The quality of predictions from the random forest models varies between them but remains high and reliable across all scenarios (Appendix 5). OOB error varies from 1.65 to 7.75%. The model predicted the location of service and agriculture firms very well (for 97%) and much worse in the production and construction sectors (for 45%). This result is consistent with the logit model results which showed strong patterns with respect to population density for agriculture and service sectors, while a bit more flexible relations were presented for the production and construction firms. Therefore, both probit and random forest models show that population density is a key factor for explaining the business location, irrespective of the differences across individual sectors.

**Elasticity model** (Eq. 11) gives unambiguous results. The elasticity of agglomeration with respect to population density is around 0.76–0.79, which is the expected magnitude (Table 4). This means that with a doubling population density, the agglomeration of firms increases by ca.76%. The coefficient below 1 is a natural consequence of congestion effects. Population and business cannot grow into infinity in highly congested areas. The reduction in available real estate that accompanies the agglomeration patterns extinguishes the multiplication effects in people–firms relations. The general pattern with regard to population is uniform—both 1st and 2nd line firms have similar values of elasticity, with a slightly stronger impact of population on the innovative companies. The elasticity of agglomeration is highly localised—the size of the city and distance to the centre matter. The model indicates a huge business potential in suburbs (up to 25 km) of almost all cities—coefficients for location dummies (*dist_…_25*) when summed up with the elasticity coefficient make the elasticity > 1, which means stronger location potential for firms than people in those areas.

**Table 4:** Elasticity model with localised effects.

Variable	Pooled data	1st line firms	2nd line firms
(Intercept)	3.27***	3.07***	3.27***
Logarithn of local population density (log_locPdens)	0.76***	0.79***	0.76***
Dummy for location in high-density cluster of firms (COREfirms)	0.11***	0.01	0.12***
Dummy for location in high-density cluster of people (COREpopul)	−0.12***	−0.03	−0.12***
Dummy for location up to 10 km to core city (dist_core_10)	0.05***	0.10***	0.05***
Dummy for location up to 10 km to midsize city (dist_midsize_10_	0.24***	−0.26***	0.24***
Dummy for location up to 10 km to regional city (dist_regional_10)	−0.43***	−0.18***	−0.44***
Dummy for location up to 10 km to local bigger city (dist_localbig_10)	−0.09***	−0.13***	−0.09***
Dummy for location up to 10 km to local small city (dist_localsmall_10)	0.13***	0.23***	0.12***
Dummy for location between 10 and 25 km from the core city (dist_core_25)	0.74***	0.58***	0.74***
Dummy for location between 10 and 25 km from the midsize city (dist_midsize_25)	0.40***	0.65***	0.40***
Dummy for location between 10 and 25 km from the regional city (dist_regional_25)	0.09***	−0.02	0.10***
Dummy for location between 10 and 25 km from the local big city (dist_localbig_25)	0.26***	0.57***	0.25***
Dummy for location between 10 and 25 km from the local small city (dist_localsmall_25)	0.19***	0.53***	0.18***
Dummy for location between 25 and 50 km from the core city (dist_core_50)	0.10***	−0.04	0.10***
Dummy for location between 25 and 50 km from the midsize city (dist_midsize_50)	−0.02***	0.40***	−0.02***
Dummy for location between 25 and 50 km from the regional city (dist_regional_50)	−0.27***	−0.66***	−0.26***
Dummy for location between 25 and 50 km from the local big city (dist_localbig_50)	0.09***	0.71***	0.08***
Dummy for location between 25 and 50 km from the local small city (dist_localsmall_50)	−0.50***	−1.02***	−0.50***
AIC	3,047,602	103,234.5	2,935,663
BIC	3,047,838	103,407.5	2,935,898
Log Likelihood	−1,523,781	−51,597.2	−1,467,811
Deviance	1,275,973	28,363.47	1,246,256
Num. obs.	983,719	42,341	941,378

P-value note: ***<0.001< **<0.01< *<0.05

## Discussion of the results and conclusions

Those empirical results confirm the need to enrich the conceptual design of business location theory by adding the impact of the population to a set of its determinants. The elasticity model that was used in our analysis supports the claim that population density can significantly stimulate firm births. This paper adds a few novelties to business location literature. First, it introduces and links the concept of economies of density. Secondly, it shows that the attraction mechanism is hierarchical and involves population and firms divided into 1st and 2nd line. Third, through advanced modelling on individual data, it is not only a theoretical hypothesis but a study with real verification of the proposed theory.

Specific conclusions that can be drawn from the study presented can have profound implications for existing research. **First**,** we show in a comprehensive way the relation between population density and business agglomeration.** We show that there exists a mechanism of attraction, which links the business activity to its root cause—population density. There is evidence that population density can be a significant factor in firms’ location, next to agglomeration. However, economies of density appear hierarchically alongside economies of agglomeration. This is not a trade-off relation—economies of density and agglomeration co-exist and affect both 1st and 2nd line companies. The attraction mechanism works on different spatial levels (on the intra-urban micro-scale as well as on the intra-regional macro-scale), making it a universal and robust phenomenon. Business sectors also differ in terms of location determinants^81^. This means there is no one-fit-all theory linking the relationship between the economies of density and the economies of agglomeration. Model results show that some firms prefer more vibrant, urban places, while others prefer more secluded, peripheral locations with lower population density, however, population density remains an important locational factor regardless of the sector.

**Secondly**,** we estimated the local elasticities.** There is an evident and stable level of elasticity (ε) of business agglomeration with respect to the residential population. As it was shown, when local population density doubles, - local business agglomeration increases on average by ca. 76% (and even 79% for 1st line firms). The limits of space, congestion and concentration effects result in an elasticity of less than 1 (ε < 1) eliminating infinite growth in the number of firms. In the most densely populated districts, more companies tend to locate in the suburbs (up to 25 km from the main city), pushing the entrepreneurial potential stimulated by population growth towards peripheral areas. This highly spatial-related effect explains why direct suburbs and industrial rings are increasingly appealing for business while being less attractive for human settlements.

**Third**,** we add an empirical verification of old dilemma Marshallian or Jacobian agglomeration externalities.** Business benefits from two density-related phenomena: density externalities (understood as the number of inhabitants in a place) and urbanization externalities (understood as a high-density clustering). There is no overlap between the two– both effects are observable in explaining firms’ location decisions. Depending on the sector, Marshallian or Jacobian agglomeration externalities can be observed. 2nd line firms often locate next to companies from the same sector. This Marshallian agglomeration effect can be explained by a path-dependent process which makes firms mimic entrepreneurs specialising in the same field, which also suggests homogeneity of (location-determining) factors for firms in a particular sector At the same time, almost all of the analysed sectors (except for 2nd line production firms) were attracted to areas with higher industrial diversity, so for both 1st line and most 2nd line firms the Jacobian type externalities matter.

**Fourth**,** the presented methodological approach builds the comprehensive micro-geography toolbox.** Quantitative methods proposed in this paper: mediation model, binary choice model and elasticity model are complementary approaches to track the analysed relations. We believe that our results open a new path for research on business location and business demography. If the effects analysed in our study are not taken into account simultaneously, it may lead to biased coefficients in business choice models and even misleading conclusions in business location research. Focusing only on agglomeration economics (as in many previous studies) makes the analysis much more limited in terms of the effects related to the spatial location of firms. Confirming that population density is one of the root determinants of firm creation, its impact should not be overlooked in business location studies.

## Electronic supplementary material

Below is the link to the electronic supplementary material.

Supplementary Material 1

## Data Availability

Representative sample of data used in this analysis is available at Figshare repository at https://figshare.com/s/c89cd82ea19ce8f3cece, while point data for population from census are available at Figshare repository at https://figshare.com/s/b0feffe666b4f4bece93.

## References

[1] Eriksson, R. H. Localized spillovers and knowledge flows: How does proximity influence the performance of plants?. *Econ. Geogr.* **87**(2), 127–152 (2011).

[2] Iammarino, S. & McCann, P. *Multinationals and economic geography: Location, technology and innovation* (Edward Elgar Publishing, 2013).

[3] Marshall, A. *Principles of Economics* (Macmillan, 1890).

[4] Fujita, M., Krugman, P. R. & Venables, A. *The spatial economy: Cities, regions, and international trade* (MIT Press, 2001).

[5] Ottaviano, G. I. & Thisse, J. F. New economic geography: what about the N?. *Environ. Plan. A* **37**(10), 1707–1725 (2005).

[6] Duranton, G. & Puga, D. Micro-foundations of urban agglomeration economies. In *Handbook of regional and urban economics* Vol. 4 2063–2117 (Elsevier, 2004).

[7] Allam, Z., Bibri, S. E., Chabaud, D. & Moreno, C. The ‘15-Minute City’concept can shape a net-zero urban future. *Human. Soc. Sci. Commun.* **9**(1), 1–5 (2022).

[8] Smętkowski, M., Moore-Cherry, N. & Celińska-Janowicz, D. Spatial transformation, public policy and metropolitan governance: secondary business districts in Dublin and Warsaw. *Eur. Plan. Stud.* **29**(7), 1331–1352 (2021).

[9] Holmes, T. J. The diffusion of Wal-Mart and economies of density. *Econometrica* **79**(1), 253–302 (2011).

[10] Braeutigam, R. R., Daughety, A. F. & Turnquist, M. A. A firm specific analysis of economies of density in the US railroad industry. *J. Ind. Econ.* **33**(1), 3–20 (1984).

[11] Caves, D. W., Christensen, L. R. & Tretheway, M. W. Economies of density versus economies of scale: why trunk and local service airline costs differ. *RAND J. Econ.* **15**, 471–489 (1984).

[12] Holmes, T. J. & Lee, S. Economies of density versus natural advantage: Crop choice on the back forty. *Rev. Econ. Stat.* **94**(1), 1–19 (2012).

[13] Nielsen, B. B., Asmussen, C. G., Weatherall, C. D. & Lyngemark, D. H. Marshall vs Jacobs agglomeration and the micro-location of foreign and domestic firms. *Cities* **117**, 103322 (2021).

[14] Ahlfeldt, G. M., Redding, S. J., Sturm, D. M. & Wolf, N. The economics of density: Evidence from the Berlin Wall. *Econometrica* **83**(6), 2127–2189 (2015).

[15] Morikawa, M. Economies of density and productivity in service industries: An analysis of personal service industries based on establishment-level data. *Rev. Econ. Stat.* **93**(1), 179–192 (2011).

[16] Roberts, M. J. Economies of density and size in the production and delivery of electric power. *Land Econ.* **62**(4), 378–387 (1986).

[17] Dubé, J., Brunelle, C. & Legros, D. Location theories and business location decision: A micro-spatial investigation in Canada. *Rev. Reg. Stud.* **46**(2), 143–170 (2016).

[18] Andersson, M., Larsson, J. P. & Wernberg, J. The economic microgeography of diversity and specialization externalities–firm-level evidence from Swedish cities. *Res. Policy* **48**(6), 1385–1398 (2019).

[19] Behrens, K., Duranton, G. & Robert-Nicoud, F. Productive cities: Sorting, selection, and agglomeration. *J. Polit. Econ.* **122**(3), 507–553 (2014).

[20] Anas, A., Arnott, R. & Small, K. A. Urban spatial structure. *J. Econ. Lit.* **36**(3), 1426–1464 (1998).

[21] Duranton, G. & Puga, D. The economics of urban density. *J. Econ. Perspect.* **34**(3), 3–26 (2020).

[22] McCann, P. & Van Oort, F. Theories of agglomeration and regional economic growth: a historical review. In *Handbook of Regional Growth and Development Theories* 6–23 (Edward Elgar Publishing, 2019).

[23] Porter, M. E. New strategies for inner-city economic development. *Econ. Dev. Q.* **11**(1), 11–27 (1997).

[24] Markusen, A. & Schrock, G. Consumption-driven urban development. *Urban Geogr.* **30**(4), 344–367 (2009).

[25] Glaeser, E. L., Kolko, J. & Saiz, A. Consumer city. *J. Econ. Geogr.* **1**(1), 27–50 (2001).

[26] Stam, E. & Van de Ven, A. Entrepreneurial ecosystem elements. *Small Bus. Econ.* **56**, 809–832 (2021).

[27] Meltzer, R. Gentrification and small business: Threat or opportunity?. *Cityscape* **18**(3), 57–86 (2016).

[28] Waldfogel, J. The median voter and the median consumer: local private goods and population composition. *J. Urban Econ.* **63**(2), 567–582 (2008).

[29] Lancaster, K. The economics of product variety: A survey. *Mark. Sci.* **9**(3), 189–206 (1990).

[30] Giuliano, G., Kang, S. & Yuan, Q. Agglomeration economies and evolving urban form. *Ann. Reg. Sci.* **63**, 377–398 (2019).

[31] Tavassoli, S. & Jienwatcharamongkhol, V. Survival of entrepreneurial firms: the role of agglomeration externalities. *Entrep. Reg. Dev.* **28**(9–10), 746–767 (2016).

[32] Zhong, T., Lü, G., Zhong, X., Tang, H. & Ye, Y. Measuring human-scale living convenience through multi-sourced urban data and a geodesign approach: Buildings as analytical units. *Sustainability* **12**(11), 4712 (2020).

[33] Marcus, L. Spatial capital. *J. Space Syntax* **1**(1), 30–40 (2010).

[34] Ewing, R. & Cervero, R. Travel and the built environment: A meta-analysis. *J. Am. Plan. Assoc.* **76**(3), 265–294 (2010).

[35] Ye, X., Tan, H., Zhang, Y., Zhang, L. & Zhang, Z. Research on convenience index of urban life based on POI data. *J. Phys. Conf. Ser.* **1646**(1), 012073 (2020).

[36] Wang, Z., Li, H. & Shang, H. Convenience analysis of public service facilities in the walking circle of urban housing estates. *J. Landsc. Res.* **13**(4), 1–4 (2021).

[37] Wu, Y., Wei, Y. D., Li, H. & Liu, M. Amenity, firm agglomeration, and local creativity of producer services in Shanghai. *Cities* **120**, 103421 (2022).

[38] Jacobs, A. & Appleyard, D. Toward an urban design manifesto. *J. Am. Plan. Assoc.* **53**(1), 112–120 (1987).

[39] Buys, L. & Miller, E. Conceptualising convenience: Transportation practices and perceptions of inner-urban high density residents in Brisbane, Australia. *Transp. Policy* **18**(1), 289–297 (2011).

[40] Howley, P., Scott, M. & Redmond, D. Sustainability versus liveability: an investigation of neighbourhood satisfaction. *J. Environ. Plan. Manag.* **52**(6), 847–864 (2009).

[41] Bramley, G., Dempsey, N., Power, S., Brown, C. & Watkins, D. Social sustainability and urban form: evidence from five British cities. *Environ. Plan. A* **41**(9), 2125–2142 (2009).

[42] Ullman, E. L. Presidential address: The nature of cities reconsidered. In *Papers of the Regional Science Association* (Vol. 9, No. 1, pp. 7–23) (Springer, 1962).

[43] Hägerstrand, T. What about people in regional science?. In *Papers of the Regional Science Association* (Vol. 24) (1970).

[44] Anderson, R., Condry, B., Findlay, N., Brage-Ardao, R. & Li, H. Measuring and valuing convenience and service quality. A review of global practices and challenges from the public transport sector(2013).

[45] Amitai, E. Normative-affective factors toward a new decision-making model. *J. Econ. Psychol.* **9**, 125–150 (1988).

[46] Kenyon, S. & Lyons, G. The value of integrated multimodal traveller information and its potential contribution to modal change. *Transport. Res. Part F* **6**, 1–21 (2003).

[47] Anable, J. & Gatersleben, B. All work and no play? The role of instrumental and affective factors in work and leisure journeys by different travel modes. *Transport. Res. Part A Policy Pract.* **39**(2–3), 163–181 (2005).

[48] Thünen, J. V. *Der isolierte Staat. Beziehung auf Landwirtschaft und Nationalökonomie* (Perthes, 1826).

[49] Alonso, W. *Location and Land Use: Toward a General Theory of Land Rent* (Harvard University Press, 1964).

[50] Camagni, R., Capello, R. & Caragliu, A. The rise of second-rank cities: what role for agglomeration economies? In *Second Rank Cities in Europe* 39–59 (2017).

[51] Camagni, R., Capello, R. & Caragliu, A. Agglomeration economies in large versus small cities: similar laws, high specifities. In *The Rise of the City* 85–114 (Edward Elgar Publishing, 2015).

[52] Camagni, R., Capello, R. & Caragliu, A. Static vs. dynamic agglomeration economies. Spatial context and structural evolution behind urban growth. *Pap. Reg. Sci.* **95**(1), 133–158 (2016).

[53] Baumont, C., Ertur, C. & Le Gallo, J. Spatial analysis of employment and population density: the case of the agglomeration of Dijon 1999. *Geogr. Anal.* **36**(2), 146–176 (2004).

[54] Ahlfeldt, G. M. & Pietrostefani, E. The economic effects of density: A synthesis. *J. Urban Econ.* **111**, 93–107 (2019).

[55] Barufi, A. M. B., Haddad, E. A. & Nijkamp, P. Urban agglomeration, city size, and spatial density effects on wage flexibility: New evidence on the wage curve in Brazil. *Reg. Sci. Policy Pract.* **15**(9), 1998–2026 (2023).

[56] Andersson, M., Klaesson, J. & Larsson, J. P. How local are spatial density externalities? Neighbourhood effects in agglomeration economies. *Reg. Stud.* **50**(6), 1082–1095 (2016).

[57] Maré, D. C. & Graham, D. J. Agglomeration elasticities and firm heterogeneity. *J. Urban Econ.* **75**, 44–56 (2013).

[58] Graham, D. J. Agglomeration, productivity and transport investment. *J. Transp. Econ. Policy (JTEP)* **41**(3), 317–343 (2007).

[59] Melo, P. C., Graham, D. J. & Noland, R. B. A meta-analysis of estimates of urban agglomeration economies. *Reg. Sci. Urban Econ.* **39**(3), 332–342 (2009).

[60] Audretsch, D. B., Cunningham, J. A., Kuratko, D. F., Lehmann, E. E. & Menter, M. Entrepreneurial ecosystems: economic, technological, and societal impacts. *J. Technol. Transfer* **44**, 313–325 (2019). 10.1007/s10961-018-9690-4 PMC6422980 30956392

[61] Yao, Y., Pan, H., Cui, X. & Wang, Z. Do compact cities have higher efficiencies of agglomeration economies? A dynamic panel model with compactness indicators. *Land Use Policy* **115**, 106005 (2022).

[62] Jacobs, J. *The Death and Life of Great American Cities* (Random House, 1961).

[63] Meltzer, R. & Schuetz, J. Bodegas or bagel shops? Neighborhood differences in retail and household services. *Econ. Dev. Q.* **26**(1), 73–94 (2012).

[64] Carpenter, C. W., Van Sandt, A. & Loveridge, S. Empirical methods in business location research. *Reg. Stud. Reg. Sci.* **8**(1), 344–361 (2021).

[65] Kubara, M. & Kopczewska, K. Akaike information criterion in choosing the optimal k-nearest neighbours of the spatial weight matrix. *Spat. Econ. Anal.* **19**(1), 73–91 (2024).

[66] Baron, R. M. & Kenny, D. A. The moderator–mediator variable distinction in social psychological research: Conceptual, strategic, and statistical considerations. *J. Pers. Soc. Psychol.* **51**(6), 1173 (1986). 3806354 10.1037//0022-3514.51.6.1173

[67] Imai, K., Keele, L. & Tingley, D. A general approach to causal mediation analysis. *Psychol. Methods* **15**(4), 309 (2010). 20954780 10.1037/a0020761

[68] Antonakis, J., Bendahan, S., Jacquart, P. & Lalive, R. On making causal claims: A review and recommendations. *Leadership Q.* **21**(6), 1086–1120 (2010).

[69] Kopczewska, K. Spatial machine learning: new opportunities for regional science. *Ann. Reg. Sci.* **68**(3), 713–755 (2022).

[70] Pan, H., Deal, B., Chen, Y. & Hewings, G. A reassessment of urban structure and land-use patterns: Distance to CBD or network-based?—Evidence from Chicago. *Reg. Sci. Urban Econ.* **70**, 215–228 (2018).

[71] Backman, M. & Karlsson, C. Location of new firms: Influence of commuting behaviour. *Growth Change* **48**(4), 682–699 (2017).

[72] Guimaraes, P., Figueiredo, O. & Woodward, D. Agglomeration and the location of foreign direct investment in Portugal. *J. Urban Econ.* **47**(1), 115–135 (2000).

[73] Elgar, I., Farooq, B. & Miller, E. J. Modeling location decisions of office firms: Introducing anchor points and constructing choice sets in the model system. *Transport. Res. Rec.* **2133**(1), 56–63 (2009).

[74] Balbontin, C. & Hensher, D. A. Firm-specific and location-specific drivers of business location and relocation decisions. *Transp. Rev.* **39**(5), 569–588 (2019).

[75] Carlino, G. A., Chatterjee, S. & Hunt, R. M. Urban density and the rate of invention. *J. Urban Econ.* **61**(3), 389–419 (2007).

[76] Klein, A. & Crafts, N. Agglomeration externalities and productivity growth: US cities, 1880–1930. *Econ. Hist. Rev.* **73**(1), 209–232 (2020).

[77] Kopczewska, K., Churski, P., Ochojski, A. & Polko, A. *Measuring Regional Specialisation: A New Approach* (Springer, 2017).

[78] Pominova, M., Gabe, T. & Crawley, A. The stability of location quotients. *Rev. Reg. Stud.* **52**(3), 296–320 (2022).

[79] Artz, G. M., Kim, Y., Orazem, P. F. & Han, P. J. Which small towns attract start-ups and why? Twenty years of evidence from Iowa. *Am. J. Agric. Econ.* **103**(2), 702–720 (2021).

[80] Porter, M. E. Clusters and competition: new agendas for companies, governments, and institutions. In *On competition* (ed. Porter, M. E.) (Harvard Business Press, 2008).

[81] Ferreira, J. J., Fernandes, C. I., Raposo, M. L., Thurik, R. & Faria, J. R. Entrepreneur location decisions across industries. *Int. Entrep. Manag. J.* **12**, 985–1006 (2016).

