# Supplementary material for: Population density as the attractor of business to the place
Source: Sci Rep. 2024 Sep 27;14:22234. doi: 10.1038/s41598-024-73341-8 (PMC11436939; doi:10.1038/s41598-024-73341-8)
Supplement: Supplementary file 1 — Supplementary Material 1 [file 41598_2024_73341_MOESM1_ESM.docx]

**Population density**

**as the attractor of business to the place**

**Appendices**

**Appendix 1: Economic sectors in REGON register**

REGON register collects information on all existing firms in Poland. Data are publicly available at https://wyszukiwarkaregon.stat.gov.pl/appBIR/index.aspx. The analysed dataset included all registered firms in 2012 in the NUTS1 Mazovian region (Poland). Sectors were coded with capital letters as follows (Tab.A1):

**Table A1: Economic sectors used in analysis**

| Symb. | Abbreviation | Full name of sector | Number of obs. |
| --- | --- | --- | --- |
| A | Agri_forest | Agriculture, forestry, hunting and fishing | 253’514 |
| B | Mining | Mining and exploration | 862 |
| C | Indust_process | Industrial processing | 58’824 |
| D | Energy | Producing and supplying in electricity, gas, steam, hot water and air conditioning systems | 1’944 |
| E | Water_waste | Water supply; wastewater management, waste management and remediation activities | 2’359 |
| F | Constr | Construction | 71’579 |
| G | Trade | Wholesale and retail trade; repair of motor vehicles and motorcycles | 191’442 |
| H | Logistics | Transportation and storage | 44’894 |
| I | Hotels_catering | Activities related to accommodation and catering services | 18’223 |
| J | Inform_communic | Information and communication | 38’822 |
| K | Finance_insur | Financial and insurance activities | 24’606 |
| L | Real_estate_serv | Activities related to real estate services | 32’056 |
| M | Prof_scien_tech_serv | Professional, scientific and technical activities | 97’762 |
| N | Administr | Administration and support service activities | 26’526 |
| O | Security_publ_adm | Public administration and defense; compulsory social security | 3’471 |
| P | Educat | Education | 26’746 |
| Q | Healt_social_assist | Healthcare and social assistance | 32’988 |
| R | Culture_leasure | Activities related to arts, entertainment and recreation | 11’847 |
| S | Other_serv | Other service activities | 45’101 |
| T | Private_act | Private households hiring employees; households producing goods and providing services for their own needs, | 5 |
| U | Org_extraterit | Organisations and extraterritorial teams | 144 |
| **TOTAL** | | | **983’715** |

This dataset includes also PKD7 (*Polska Klasyfikacja Dzialalności*) five digit code which describes the major declared activity of a given firm. The analysed dataset included 646 individual PKD7 activities out of 654 available PKD7 codes.

PKD7 codes can be classified according to technology involvement. European classification (Eurostat, 2020) is based on NACE codes. Polish PKD7 classification is linked to NACE. Polish Agency for Enterprise Development (PARP, *Polska Agencja Rozwoju Przedsiębiorczości*) publishes PKD7 codes which are typical for business activities involving medium-high-technology, high-technology or high-tech knowledge-intensive services). This classification was used to distinguish high-tech firms. Data was recoded using Polish PKD classification being equivalent to NACE classification.

REGON dataset includes also information on employment size. Variable *Gr_empl* reports five employment classes: class 1 for up to 9 persons, class 2 for 10-49 persons, class 3 for 50-249 persons, class 4 for 250-1000 persons and class 5 for more than 1000 persons. Variable *Empl* gives the approximate mid-value of group: 5 for class 1, 30 for class 2, 150 for class 3, 600 for class 6 and 1500 for class 5.

Labelling as KIBS (Knowledge-Intensive Business Services) was based on wide classification following [Wyszkowska-Kuna, 2016, Tab.2.2] which includes section K (financial and insurance activities) group 64 (financial service activities, except insurance and pension funding), group 65 (insurance, reinsurance and pension funds pensions, excluding compulsory social security), group 66 (activities auxiliary to financial services and insurance and pension funding) and section M (professional, scientific and technical activities) group 70 (activities of head offices; management consultancy activities), group 71 (architectural and engineering activities; technical testing and analysis), group 72 (research and development), group 73 (advertising, market research and public opinion polling) and group 74 (other professional, scientific and technical activities).

**Appendix 2: Location of regional cities and DBSCAN clusters**

Relative location to cities was derived using real locations of 39 cities in the Mazovian NUTS1 region (*województwo mazowieckie*), Poland (Tab.A2). They were grouped into 5 population size: core 1mln+ (1 city, Warsaw), midsize 100K+ (2 cities, Radom and Płock), regional 50K+ (4 cities, Siedlce, Pruszków, Legionowo, Ostrołęka), local big 30K+ (9 cities), local small 15-30K (23 cities). Each firm was checked if it is located in a radius of 10, 25 and 50 km from the centre of each city. Finally, instead of distances to particular cities, the dataset included 15 dummy variables: *dist_core_10, dist_core_25, dist_core_50, dist_midsize_10, dist_midsize_25, dist_midsize_50, dist_regional_10, dist_regional_25, dist_regional_50, dist_localbig_10, dist_localbig_25, dist_localbig_50, dist_localsmall_10, dist_localsmall_25, dist_localsmall_50*.

**Table A2: Cities located in NUTS1 Mazovian region included in the analysis**

| ID | city | Population (persons) | area (km2) | Density | longitude | latitude |
| --- | --- | --- | --- | --- | --- | --- |
| 1 | Warszawa | 1’729’119 | 517.9 | 3339 | 21.06119 | 52.23294 |
| 2 | Radom | 217’834 | 111.8 | 1948 | 21.01389 | 51.4152 |
| 3 | Płock | 122’572 | 88.06 | 1392 | 19.64501 | 52.53549 |
| 4 | Siedlce | 76’585 | 31.87 | 2403 | 22.14144 | 52.16167 |
| 5 | Pruszków | 59’796 | 19.19 | 3116 | 20.66378 | 52.17202 |
| 6 | Legionowo | 54’246 | 13.6 | 3989 | 20.90458 | 52.40488 |
| 7 | Ostrołęka | 52’792 | 29 | 1820 | 21.51907 | 53.07745 |
| 8 | Piaseczno | 45’270 | 16.22 | 2791 | 20.88196 | 52.07337 |
| 9 | Otwock | 45’073 | 47.33 | 952 | 21.21945 | 52.11633 |
| 10 | Ciechanów | 44’673 | 32.84 | 1360 | 20.54715 | 52.87107 |
| 11 | Żyrardów | 41’056 | 14.35 | 2861 | 20.40536 | 52.05526 |
| 12 | Mińsk Mazowiecki | 40’028 | 13.12 | 3051 | 21.52169 | 52.17917 |
| 13 | Wołomin | 37’418 | 17.24 | 2170 | 21.20118 | 52.34264 |
| 14 | Sochaczew | 37’333 | 26.13 | 1429 | 20.1092 | 52.23635 |
| 15 | Ząbki | 32’376 | 11.13 | 2909 | 21.07776 | 52.29252 |
| 16 | Mława | 30’893 | 35.5 | 870 | 20.31333 | 53.13291 |
| 17 | Grodzisk Mazowiecki | 29’988 | 13.19 | 2274 | 20.59806 | 52.10513 |
| 18 | Marki | 29’395 | 26.03 | 1129 | 21.04995 | 52.33716 |
| 19 | Nowy Dwór Mazowiecki | 28’361 | 28.27 | 1003 | 20.42122 | 52.43413 |
| 20 | Wyszków | 27’205 | 20.78 | 1305 | 21.41228 | 52.5923 |
| 21 | Piastów | 22’862 | 5.76 | 3969 | 20.77894 | 52.18553 |
| 22 | Ostrów Mazowiecka | 22’770 | 22.09 | 1031 | 21.85631 | 52.80713 |
| 23 | Płońsk | 22’435 | 11.6 | 1934 | 20.32957 | 52.62666 |
| 24 | Kobyłka | 21132 | 19.64 | 1076 | 21.16377 | 52.34008 |
| 25 | Józefów | 20013 | 23.92 | 837 | 21.08495 | 52.12857 |
| 26 | Sulejówek | 19’385 | 19.31 | 1004 | 21.24671 | 52.24465 |
| 27 | Pionki | 19’286 | 18.34 | 1052 | 21.41105 | 51.47252 |
| 28 | Pułtusk | 19’229 | 23 | 836 | 21.04836 | 52.70321 |
| 29 | Gostynin | 19’026 | 32.4 | 587 | 19.33104 | 52.42421 |
| 30 | Sokołów Podlaski | 18’730 | 17.5 | 1070 | 22.19902 | 52.41285 |
| 31 | Sierpc | 18’468 | 18.6 | 993 | 19.63032 | 52.85337 |
| 32 | Kozienice | 18’150 | 10.45 | 1737 | 21.50136 | 51.58732 |
| 33 | Zielonka | 17’434 | 79.48 | 219 | 21.07706 | 52.293 |
| 34 | Konstancin-Jeziorna | 17’371 | 17.74 | 979 | 21.07058 | 52.08389 |
| 35 | Przasnysz | 17’337 | 25.16 | 689 | 20.84989 | 53.01855 |
| 36 | Garwolin | 17’160 | 22.08 | 777 | 21.58779 | 51.89536 |
| 37 | Łomianki | 16’632 | 8.4 | 1980 | 20.85897 | 52.33345 |
| 38 | Grójec | 16’430 | 8.57 | 1917 | 20.83774 | 51.86819 |
| 39 | Milanówek | 16’427 | 13.52 | 1215 | 20.63528 | 52.12485 |

**Figure A1: Urban settlement in Mazovian region**


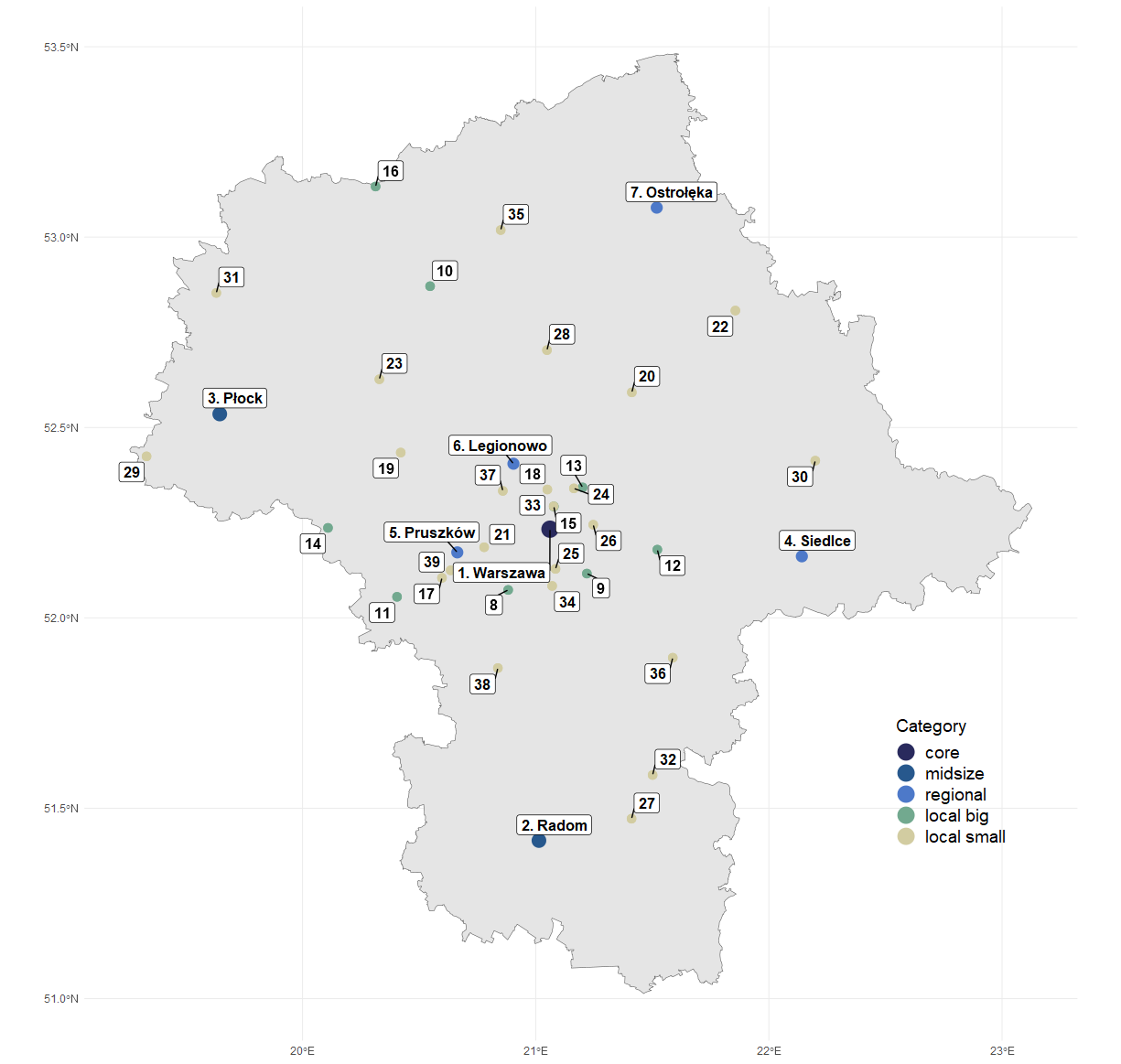


**DBSCAN algorithm** is density-based clustering. It divides geo-localised points into core points (located in high-density areas) and noise (located in low-density areas) – there is no pre-assumption on a number of clusters. The clustering mechanism uses for each point a circle of specified radius and checks how many points are in this circle – if the number of points exceeds the assumed threshold, the given location is classified as core, if not – as noise. In this study, we took a radius of 0.03° what is equivalent to ca.3.3 km and minPts=75 firms / 500 persons. The selection of these parameters involved a deeper analysis of the stability of the solution. An alternative way to DBSCAN could be setting a threshold for the local population or business density and delineating locations belonging to high-density clusters. These methods can be applied to regional or grid data, while their main disadvantages remain all problems linked to data aggregation, including MAUP (Modified Areal Unit Problem).

**Figure A2: Spatial data used in analysis: a) business location with DBSCAN high-density clusters, b) population location with DBSCAN high-density clusters**


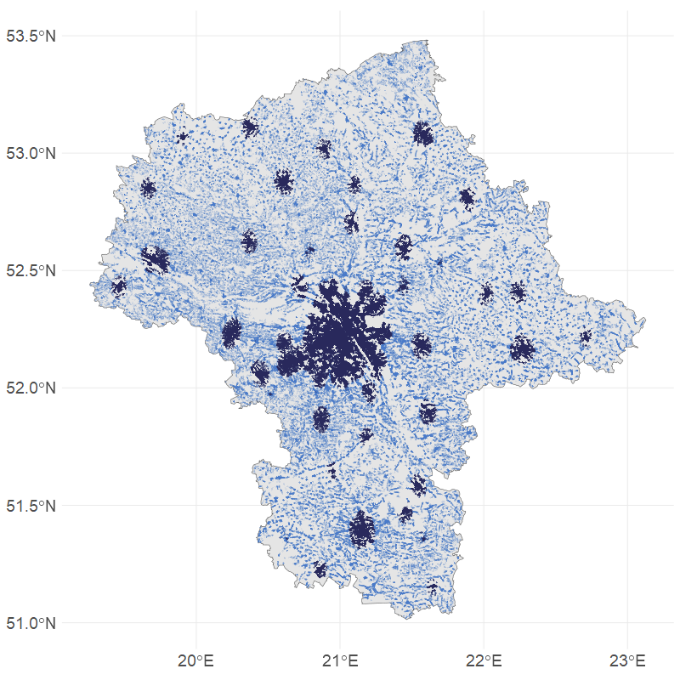

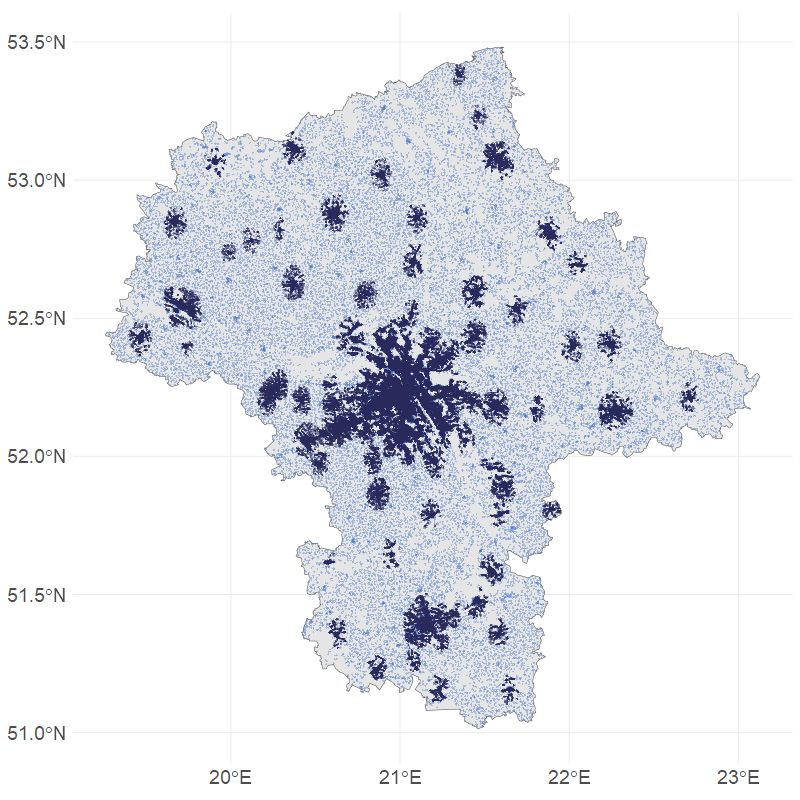


**Appendix 3: Spatial granulation – justification of circles and grid-to-point disaggregation**

**Using a circle as a bounding box** for the spatial neighbourhood is well justified with other spatial methods also based on circles. One should mention here: i) DBSCAN, which is testing how many points are in a specified radius to detect high-density areas (Ester et al., 1996), ii) Geographically Weighted Regression (GWR) which performs local regressions in optimised bandwidth (radius) (Brunsdon et al., 1998), iii) Ripley’s K which outputs the agglomeration function by counting points in increasing radii, iv) SPAG which calculates area covered by overlapping circles representing points to measure agglomeration synthetically (Kopczewska et al., 2019), v) Kuldorf’s spatial scan statistics which compares within a moving ring the probability of being the case given populations at risk inside and outside the ring (Kulldorff, 1997). This popularity of circular neighbourhoods results from their natural properties. Let’s compare the circle with the commonly used grid neighbourhood in a queen form (all eight surrounding boxes neighbouring with a selected one). Assuming that the areas of 9 squares (9*side^2^) and a single circle (π*r^2^) are equal, the radius of the circle equals 1.69 of the single square’s side (Fig.A3). This matters for the relation of the maximum distance between the core and the most extreme point. In the radial setting, maximum distance equals r (1.69*side), while in the squared setting, it is 3*side*$\sqrt{2}$ /2=2.12*side, what makes maximum distance in squared setting longer by 25%. Thus, with the same “catchment” capacity in a squared setting (the same area of analysis), the distances are longer which makes the potential distance-decaying pattern more intensively represented. This suggests that the power of spatial interactions is more diversified in squared data, thus, modelling may be less sensitive. This leads to the conclusion that, if possible, a radial approach should be used to deal with more coherent data.

**Figure A3: Visualisation of the distances in radial and squared approaches**


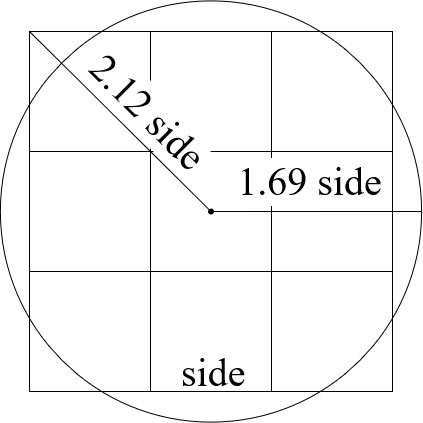


Population data, available as grid data, were disaggregated into point data. The sampling procedure assumed drawing for each grid cell (1 km x 1 km) from random spatial distribution the equivalent number of points (1 point = 100 persons) (Fig.A4). Those random points were geo-localised (longitude/latitude) and treated as point pattern. This newly created dataset was used in a few computations:

- To detect DBSCAN clusters with a high-density of population
- To find local agglomeration of population, by counting the number of inhabitants in a radius of 500 m from a given firm

**Figure A4: Creating population point pattern from grid census data**


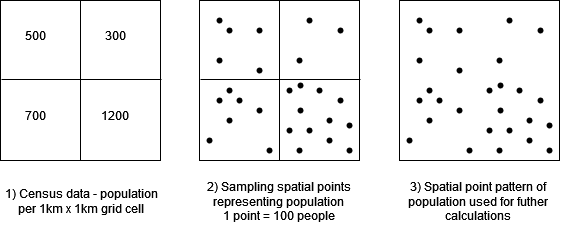


**Appendix 4: Details of probit, random forest and multidimensional scaling**

Selection of binary-choice model over e.g. count models has a methodological justification. Many studies (e.g. Carpenter et al., 2021) use count data as dependent variable to express the popularity of given surrounding (box, circle) for business location. This approach changes the research question – from location decision to determinants of clusters; and estimation method – from binary models to count models.

**Probit model** is a classical econometric solution for the binary dependent variable. It models the probability of success – that an event occurs. Probit beta coefficients show an impact of unitary change of the explanatory variable (*ceteris paribus*) on the probability that an event appears – an increase in variables with positive coefficients increases the probability of the event. Model quality can be measured in absolute terms with McFadden R^2^ (interpreted as a classical one, the higher the better, value close to 1 for a perfect fit) and relatively, between the models, with AIC (Akaike Information Criterion) (the smaller the better, AIC depends on a number of observations). Even if probit is less restrictive (than e.g. logit) regarding the normality of variables’ distributions, it has the disadvantage of being a linear combination of variables. Therefore the cross-check is being done with a supervised machine learning model – random forest, which is more flexible in the case of non-linear relationships and models inhomogeneities much better.

**Random forest** model is a well-established supervised machine learning method, being a mixture of decision tree and bootstrap. It can also be applied in spatial modelling (Kopczewska, 2022). It samples observations and variables in each iteration (tree) to find the best cut-off points of the explanatory variables in order to get coherent groups indicated by binary levels of the dependent variable. The aggregation of bootstrap results from random forest ensemble is by majority voting (bagging). The output of random forest is variable importance – information on how much the variable matters in the model. Two criteria may be used: misclassification rate, which measures the ratio of miss-classified predictions when the variable of interest has been shuffled (randomised); or decrease in node purity, which measures how much MSE (*mean square error*) is reduced when a variable of interest becomes a node split of the tree. Both criteria are measured across all trees: misclassification as a ratio of wrong predictions, and node purity as a total reduction of MSE. In both cases, the higher the indicator, the more important the variable is in predictions. For multiple models, variable importance can be reported as box plots (instead of bar plots). The quality of the random forest is typically assessed with confusion matrix (CM) and out-of-bag (OOB) prior error rate. A confusion matrix (CF) is a two-dimensional contingency table, which counts observed and predicted labels (0 and 1). Classification errors in CM (for label=0 and label=1) measure the fit misclassification ratio:

| ${err}_{obs=0}=\frac{{count}_{obs=0,fit=1}}{{count}_{obs=0,fit=0}+{count}_{obs=0,fit=1}}$ | (A.1) |
| --- | --- |

and

| ${err}_{obs=1}=\frac{{count}_{obs=1,fit=0}}{{count}_{obs=1,fit=1}+{count}_{obs=1,fit=0}}$ | (A.2) |
| --- | --- |

The lower the error the better the predictions from the model. Out-of-bag data are observations not included in the estimation of a given tree (due to bootstrap sampling), which are used for predictions of a given tree. OOB prior error rate is the misclassification rate of OOB data (it plays the role of randomised cross-validation) – the smaller the error the better.

**Multidimensional scaling** – projects k-dimensional data into n-dimensional points (n<k), mostly in form of 2D plot. It uses Kruskal scaling (1964) to calculate the Stress function expressed as:

| $STRESS=\sqrt{\frac{\sum\left( D_{ij}-d_{ij} \right)^{2}}{\sum\left( D_{ij} \right)^{2}}}$ | (A.3) |
| --- | --- |

where *i and j* are iteration operators $i,j\epsilon\left[ 1,k \right]$ (to iterate by all pairs of variables), D_ij_ are the distances in original *k* dimensions between variables *i* and *j*, and d_ij_ are distances in a new (2D) dimensions. Outcomes are 2D locations of points which illustrate the original distances best.

It can deal with any type of data, also with dummy variables by using appropriate distance metrics. One of the solutions is to use Gower distance which can cope with mixed data types – continuous and binary variables together. The optimisation algorithm finds locations of points in 2D space to minimise the Stress function, which expresses the difference between original and new distance relations. Its main advantages are flexibility due to types of data, joint (rather than pair by pair) analysis of variables, and efficient visualisation.

**Appendix 5: Empirical results of data analysis**

All computations were made in R software. For processing of geo-localised data we used {sp}, {rgeos}, {spdep}, {spatstat}, {rgdal} and {sf} packages. Density clusters and neighbourhood locations were found with {dbscan} and {nabor}. Multidimensional scaling was made with {smacof}. The modelling used {abdrf} for the random forest, {lavaan} and {mediation} for the mediation model and {ivreg} for the mediation model with instrumental variables. For synthetic outputting, we applied {stargazer} and {texreg}. Parallel computations were executed with {doParallel}.

Tab.A3 presents the descriptive statistics of the analysed dataset – for pooled data and in the division for 1^st^ line and 2^nd^ line firms. For pooled data, we present mean, standard deviation, and extreme values (minimum and maximum). For data in groups we present average values only – extreme values were similar, while the variance in subgroups was quite narrow.

**Table A3: Descriptive statistics of the dataset**

|  |  | All firms | | | |  | 1st line | 2nd line firms | | | |
| --- | --- | --- | --- | --- | --- | --- | --- | --- | --- | --- | --- |
|  |  | pooled data | | | |  |  | agricul-ture | produ-ction | constru  -ction | service |
|  | no of obs 🡪 | 983 719 | | | |  | 42 341 | 253 514 | 55 969 | 71 579 | 560 312 |
|  | Statistic | mean | st.dev | min | max |  | mean | mean | mean | mean | mean |
| neighbourhood | locPdens | 82.88 | 90.12 | 0.00 | 376.00 |  | 126.14 | 15.05 | 81.21 | 86.64 | 110.42 |
|  | locAggAgri | 86.04 | 146.41 | 0.00 | 1.91 |  | 78.89 | 86.63 | 78.74 | 84.78 | 87.20 |
|  | locAggProd | 105.62 | 133.19 | 0.00 | 865.00 |  | 160.21 | 19.83 | 110.66 | 110.23 | 139.22 |
|  | locAggConstr | 118.67 | 145.54 | 0.00 | 876.00 |  | 178.85 | 23.85 | 119.95 | 125.54 | 156.02 |
|  | locAggServ | 1 297 | 1 761 | 0.00 | 11 537 |  | 2 055 | 202.46 | 1 268 | 1 310 | 1 736 |
|  | locBIG | 13.83 | 24.69 | 0.00 | 174.00 |  | 21.85 | 1.86 | 13.68 | 13.76 | 18.67 |
|  | locHH | 0.00 | 0.0001 | 0.00 | 0.07 |  | 0.00 | 0.00 | 0.00 | 0.00 | 0.00 |
|  | locLQ | 1.54 | 0.90 | 0.00 | 5.00 |  | 1.59 | 2.16 | 1.31 | 1.10 | 1.34 |
|  | COREfirms | 0.69 | 0.46 | 0.00 | 1.00 |  | 0.94 | 0.21 | 0.78 | 0.77 | 0.87 |
|  | COREpopul | 0.75 | 0.43 | 0.00 | 1.00 |  | 0.97 | 0.31 | 0.85 | 0.84 | 0.91 |
| location | dist_core_10 | 0.33 | 0.47 | 0.00 | 1.00 |  | 0.57 | 0.03 | 0.33 | 0.34 | 0.45 |
|  | dist_midsize_10 | 0.03 | 0.17 | 0.00 | 1.00 |  | 0.02 | 0.02 | 0.04 | 0.04 | 0.03 |
|  | dist_regional_10 | 0.08 | 0.27 | 0.00 | 1.00 |  | 0.08 | 0.05 | 0.10 | 0.09 | 0.09 |
|  | dist_localbig_10 | 0.34 | 0.47 | 0.00 | 1.00 |  | 0.45 | 0.10 | 0.38 | 0.37 | 0.43 |
|  | dist_localsmall_10 | 0.54 | 0.50 | 0.00 | 1.00 |  | 0.78 | 0.21 | 0.59 | 0.59 | 0.67 |
|  | dist_core_25 | 0.53 | 0.50 | 0.00 | 1.00 |  | 0.84 | 0.09 | 0.59 | 0.57 | 0.69 |
|  | dist_midsize_25 | 0.09 | 0.28 | 0.00 | 1.00 |  | 0.04 | 0.11 | 0.11 | 0.09 | 0.07 |
|  | dist_regional_25 | 0.53 | 0.50 | 0.00 | 1.00 |  | 0.72 | 0.22 | 0.56 | 0.56 | 0.65 |
|  | dist_localbig_25 | 0.66 | 0.47 | 0.00 | 1.00 |  | 0.91 | 0.31 | 0.71 | 0.71 | 0.79 |
|  | dist_localsmall_25 | 0.84 | 0.37 | 0.00 | 1.00 |  | 0.96 | 0.66 | 0.87 | 0.87 | 0.90 |
|  | dist_core_50 | 0.64 | 0.48 | 0.00 | 1.00 |  | 0.90 | 0.27 | 0.70 | 0.70 | 0.78 |
|  | dist_midsize_50 | 0.16 | 0.37 | 0.00 | 1.00 |  | 0.05 | 0.29 | 0.16 | 0.15 | 0.11 |
|  | dist_regional_50 | 0.80 | 0.40 | 0.00 | 1.00 |  | 0.94 | 0.60 | 0.81 | 0.82 | 0.87 |
|  | dist_localbig_50 | 0.82 | 0.39 | 0.00 | 1.00 |  | 0.95 | 0.63 | 0.83 | 0.84 | 0.88 |
|  | dist_localsmall_50 | 0.99 | 0.10 | 0.00 | 1.00 |  | 1.00 | 0.97 | 0.99 | 0.99 | 1.00 |

Tab.A4 gives the details of the quality assessment for the Random Forest model. It reports the confusion matrix (CM) for 5 models. Classification errors were calculated as described in Appendix 4.

**Table A4: Quality metrics of random forest models based on confusion matrix (CM)**

|  | Model | Label | CM  0 observed | CM  1 observed | Classification error | Out-of-bag (OOB) prior error rate |
| --- | --- | --- | --- | --- | --- | --- |
| 1^st^ line | 1^st^ line firms | CM_0_predicted | 940’597 | 781 | 0.0008 | 0.0371 |
|  |  | CM_1_predicted | 35’732 | 6’609 | 0.8439 |  |
|  |  |  |  |  |  |  |
| 2^nd^ line | Agriculture | CM_0_predicted | 679’408 | 8’456 | 0.0122 | 0.0163 |
|  |  | CM_1_predicted | 6’920 | 246’594 | 0.0272 |  |
|  |  |  |  |  |  |  |
|  | Production | CM_0_predicted | 884’935 | 474 | 0.0005 | 0.0367 |
|  |  | CM_1_predicted | 34’065 | 21’904 | 0.6086 |  |
|  |  |  |  |  |  |  |
|  | Construction | CM_0_predicted | 869’212 | 587 | 0.0006 | 0.0425 |
|  |  | CM_1_predicted | 39’453 | 32’126 | 0.5511 |  |
|  |  |  |  |  |  |  |
|  | Service | CM_0_predicted | 321’286 | 59’780 | 0.1568 | 0.0800 |
|  |  | CM_1_predicted | 15’569 | 544’743 | 0.0277 |  |

**Appendix 6: Endogeneity and causality – mediation model with instrumental variables**

As stated in the text, the mediation model was applied to examine the hierarchical impact of population density on business agglomeration. The direct impact on 1^st^ line firms and the indirect impact via 2^nd^ line firms were analysed, and it was concluded that this mechanism indeed exists and mediation occurs. However, one ambiguity remained regarding the potential backward direct relation in which the 1^st^ line firms attract the population.

To address this issue, a two-step methodology (two-stage model) was employed (Fig.A5). The first stage tests the potential endogeneity between firms and the population with a standard instrumental variable (IV) approach – this is to asses any potential feedback loops. It examines the unmediated relations in two separate IV equations and evaluates the endogeneity on both the 1^st^ line and 2^nd^ line firms. This procedure was carried out with a standard IV methodology using 2SLS estimation. The tested relations are illustrated in eq.1a and eq.2a and were augmented with the IV relation (eq.1b and eq.2b).

| ${1stLine}_{i}=\alpha_{0}+a\cdot{population}_{i}+\varepsilon_{i}$  ${population}_{i}=d_{0}+d\cdot{instrument}_{i}+\varepsilon_{i}$ | (1a) (1b) |
| --- | --- |
| ${2ndLine}_{i}=b_{0}+b\cdot{population}_{i}+\varepsilon_{i}$  ${population}_{i}=e_{0}+e\cdot{instrument}_{i}+\varepsilon_{i}$ | (2a) (2b) |

The second stage links the mediation model and instrumental variables (IV), following the method by Dippel et al. (2020) to validate the robustness of the findings. This entailed testing the model with (eq.3) and without additional IV control (eq.4). This approach requires the inclusion of an instrumental variable that is correlated with the explanatory variable but not with the dependent variable. In this case, a historical IV was used – information from the past that holds the mentioned relations with variables in the model. A comprehensive discussion on the selection of instruments can be found in Puga (2010).

| ${2ndLine}_{i}=\alpha_{0}+a\cdot{population}_{i}+d\cdot{instrument}_{i}+\varepsilon_{i}$  ${1stLine}_{i}=a_{1}+c\cdot{population}_{i}+b\cdot{2ndLine}_{i}+d\cdot{instrument}_{i}+\varepsilon_{i}$ | (3) |
| --- | --- |
|  |  |
| ${2ndLine}_{i}=\alpha_{0}+a\cdot{population}_{i}+\varepsilon_{i}$  ${1stLine}_{i}=a_{1}+c\cdot{population}_{i}+b\cdot{2ndLine}_{i}+\varepsilon_{i}$ | (4) |

**Figure A5. Structure of the endogeneity testing – IV regression of population and firms, and two mediation models – with and without the additional control from the instrumental variable**


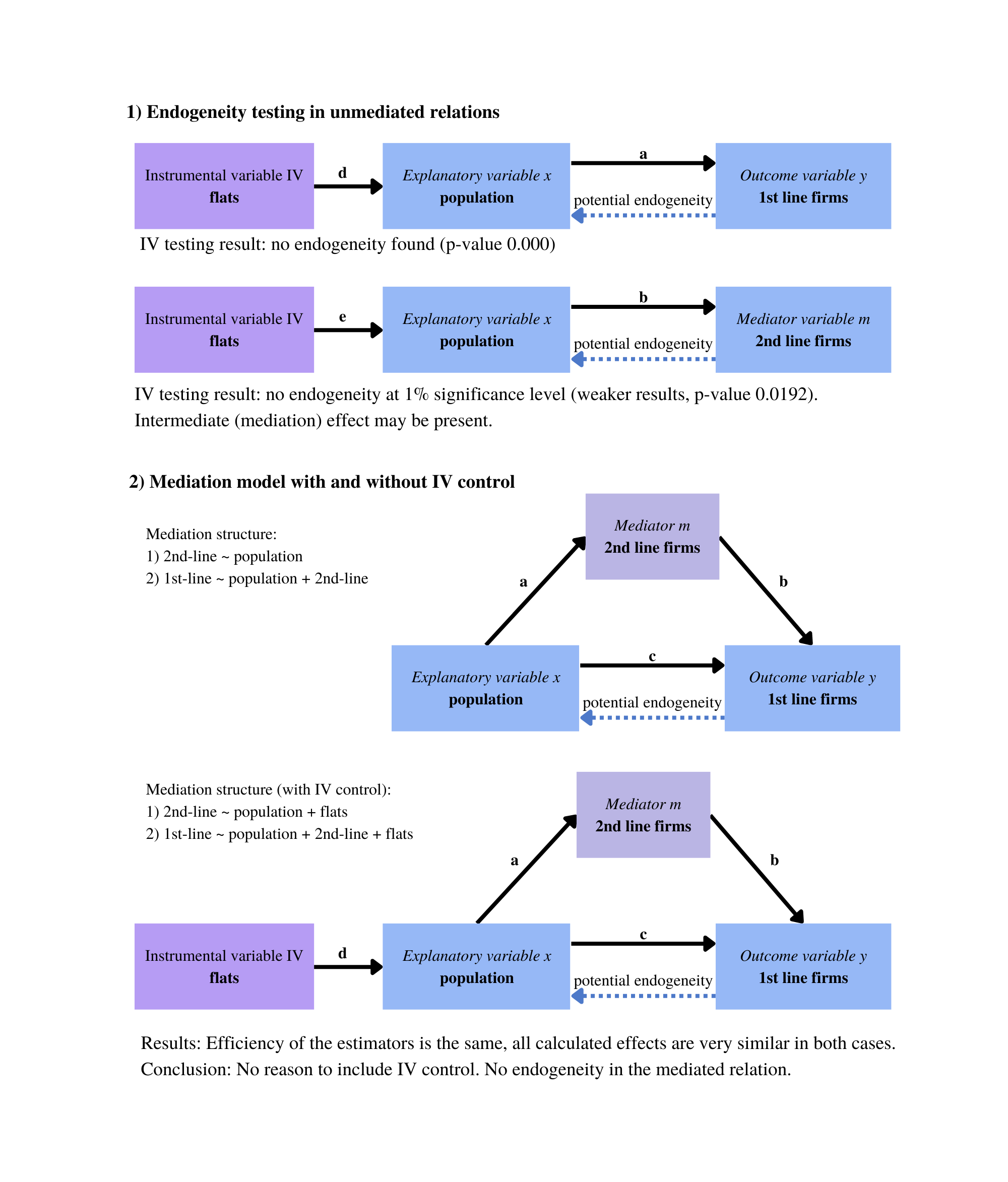


An issue of spatial granulation was encountered in the analysis, as the main study was conducted on individual geo-located point data. However, in the past, such detailed statistical information was not collected, and the lowest aggregation level is a municipality. In Poland, LAU2 is the lowest aggregation with available historical data and the only level that is comparable over time. In 1999 Poland undergone deep territorial reform as the part of European Union accession – this harmonization of territorial division of the country was to adjust the statistical reporting to rules set by EUROSTAT. LAU2 unit (municipalities, *pl gmina*) were kept fixed as they were, while other upper levels of territorial division (LAU1, NUTS3, NUTS2, NUTS1) were newly introduced. Number of regions decreased from 49 to 16. Therefore, the IV mediation regression, which includes historical data, was conducted on LAU2 rather than on individual points. This approach also allows for the generalisation of the studied mechanism, as it covers the whole territory of the country (ca. 2500 LAU2 units).

The instrument chosen to test for backward causality is the number of flats available in 1988 at the municipality level – this is an exclusion restriction. This variable is well-connected to the current population and has no relation to a current business location. The high correlation (0.98) between the flats available in the 1988 and 2021 censuses and the population registered in the census ensures that the lodgings build in the past set the trends for the modern housing market in Poland. Moreover, the 1988 data comes from the last national census organized under the communist system, making the housing system unrelated to the later introduced free market while still ensuring data reliability (the raw calculations from the 1988 census were reviewed again in 1998 after the independent Main Statistical Office was reinstated in Poland). The remaining variables used to test endogeneity follow the structure of the main study, with the difference that here the data is aggregated at the municipality level and compares the current values with the historical instrument. The 1^st^ line firms variable is measured as the number of companies operating within knowledge-intensive sectors at LAU2 in 2021. The 2^nd^ line firms variable is, respectively, measured as the number of companies specialising in retail, catering and less-productive services (2^nd^ line firms) in 2021. Population data is taken from the recent census (2021) and measured at the LAU2 level. All variables used for endogeneity testing were transformed using the logarithmic function.

Two IV regressions were performed to statistically test whether there is endogeneity between 1^st^ or 2^nd^ line firms and the population (Fig.A5). The results showed a positive and significant relationship between both types of firms and the population, with the number of flats serving as a strong instrument, confirming the validity of the chosen IV (Tab.A5). However, the endogeneity testing was not straightforward, and the interpretation is unclear for the 1^st^ line firms. While the Wu-Hausman test did not reject the null hypothesis of no endogeneity between 2^nd^ line firms and the population density, a higher significance level might suggest the presence of some hidden endogeneity between the population and 1^st^ line firms. In other words, there is no endogeneity in the relationship between population and 2^nd^ line firms, but in the case of 1^st^ line business, the hypothesis can be rejected at a higher level of statistical significance than 1%. This result does not contradict the logic presented in the main paper and suggests that there might be a hidden factor (or a mediator) that should be considered in the relationship between innovative companies and population density – the 2^nd^ line business.

**Table A5. Instrumental variable regressions results**

| **1^st^-line firms** | | | |
| --- | --- | --- | --- |
| **Coefficients** | **Estimate** | **Std. Error** | **p-value** |
| (Intercept) | -8.37302 | 0.10494 | 0.0000 |
| log(population) | 1.50689 | 0.01225 | 0.0000 |
| **Tests** | **Df1 / Df2** | **Statistic** | **p-value** |
| Weak instruments | 1 / 2374 | 16009.727 | 0.0000 |
| Wu-Hausman | 1 / 2473 | 5.491 | 0.0192 |
| **2^nd^-line firms** | | | |
| **Coefficients** | **Estimate** | **Std. Error** | **p-value** |
| (Intercept) | -4.689873 | 0.072406 | 0.0000 |
| log(population) | 1.192730 | 0.008454 | 0.0000 |
| **Tests** | **Df1 Df2** | **Statistic** | **p-value** |
| Weak instruments | 1 2374 | 16009.727 | 0.0000 |
| Wu-Hausman | 1 2473 | 0.007 | 0.9360 |

The mediation analysis was repeated on the aggregated data to verify if the conclusions obtained in the main study can be generalized for the whole country data. The mediation model was estimated in two versions – with and without the IV control – and the efficiency of the estimators was compared to show if the additional IV control is necessary (Fig.A5). The results of the standard (non-IV) mediation model run of LAU2 data show that all tested effects are highly significant (Tab.A6 – estimates without IV). Strong and positive direct and total effects indicate the causal impact of population density on the emergence of 1^st^ line firms. This impact is mediated by the presence of 2^nd^ line firms, which account for 72% of the total effect. The relationship between population density and 1^st^ line firms is again significant and positive. The results obtained with the mediation model on LAU2 data support our claim that population density is a strong predictor for the emergence of innovative businesses, both directly and via the mediation effect of less-productive service and retail companies. These results are highly comparable to the main study conducted on individual geo-located point data presented in the paper.

**Table A6. Mediation models on aggregated data – with and without IV control**

| **Effect** | **Estimate without IV** | **Estimate with IV** | **% difference** |
| --- | --- | --- | --- |
| **ACME** | 1.09336***  [1.04194; 1.15218] | 1.09526***  [1.02509; 1.16111] | - 0.17% |
| **ADE** | 0.42444***  [0.36282; 0.47546] | 0.49212***  [0.42293; 0.57061] | - 15.95% |
| **Total Effect** | 1.51779***  [1.49457; 1.53821] | 1.58738***  [1.52996; 1.63923] | - 4.58% |
| **Proportion mediated** | 0.72116***  [0.68718; 0.76006] | 0.69019***  [0.64575; 0.73052] | 4.29% |
| Significance codes: 0 ‘***’ 0.001 ‘**’ 0.01 ‘*’ 0.05 ‘.’ 0.1 ‘ ’ 1 | | | |

A comparison of the results from both mediation models (with and without IV) was conducted to ensure that the conclusions remain the same regardless of the inclusion of the instrumental variable. The estimated effects are presented in Tab.A6, which shows that the coefficients remained almost the same in both models. The estimation did not gain efficiency when the additional control was included, which proves that there is no significant endogeneity in the hierarchical relation between population density and the number of 1^st^ line firms, mediated by the 2^nd^ line firms.

**Table A7. Sensitivity analysis of both mediation models**

| **Sensitivity score** | **Model without IV** | **Model with IV** |
| --- | --- | --- |
| **Rho at which ACME=0** | 0.6000 | 0.6000 |
| **Rho at which ADE = 0** | -0.3000 | -0.3000 |

Additional support to this claim is provided by the results of the sensitivity analysis (Imai et al. 2010) for the mediation models, which were run for both IV and non-IV models. The sensitivity parameters are the same in both cases, with *rho* for the Average Causal Mediation Effect (ACME) being 0.6, and for the Average Direct Effect (ADE) rho equals -0.3 (Tab.A7). Both show considerable parameter switches necessary to overturn the conclusions shown in the model. This observation, together with the narrow confidence intervals for the estimated effects, gives us reason to trust the reasoning presented here.

To explain the emergence of 1^st^ line firms in a given area, one should take into consideration the local population density and the mediation effect of the 2^nd^ line firms. The emergence of 1^st^ line firms is caused by the local population density, and the effect is mediated by the intermediate variable, which is the number of 2^nd^ line firms operating nearby. If the mediation effect is taken into consideration, the population effect is not endogenous to the model. These two variables are enough to assert the direction of impact – local population density is the cause of innovative companies’ location.

**Appendix 7: Alternative classifications of firms**

Literature offers few classifications of firms, however, they do not fit the presented concept due to no reference to technology or spatial location. One can mention few:

basic and non-basic firms (basic firms making their incomes with external customers, while non-basic earning money on residents) – it does not deal with technology, as the criterion is source of income

tradable and non-tradable jobs/sectors (non-tradable jobs generate service consumed in place of creation, while tradable jobs generate products that can be shipped) – it does not deal with technology

high-tech knowledge-intensive firms and low-tech low-knowledge-intensive firms – even if it deals with technology, classification does not account for daily needs

"Schumpeterian firms" (disruptive firms) and "Kirznerian firms" (equilibrating firms) (Langlois, 2002) – requires not straightforward analysis of opportunities on disruption, information needs, innovativeness, commonness, and discoveries (de Jong & Marsili, 2015)

"high-end" and "low-end" firms – no middle firms

**Appendix 8: Sensitivity analysis of probit models**

The paper presents the best forms of econometric models. However, the reader may be interested in the sensitivity of the models to the inclusion or exclusion of key variables of interest. The table below shows the models for the first set of firms with alternative specifications. The first column reports the correct model as in the text. The second model shows the model without variables expressing the agglomeration of firms - it shifts the effects to population density and large firms. The third model adds non-linearity by introducing squared values of population density. It confirms the slight non-linearity, hence the random forest models presented in the text. The squared population density included in the model increases the coefficient on population density, which is typical for this type of pattern. The fourth model eliminates both variables related to population density to check how much this information affects the estimates. It biases the variables related to agglomeration (especially the dummy for high-density business clusters) and overestimates the importance of distance to cities of different sizes. The conclusion from this sensitivity analysis is that population factors should not be omitted in business location models.

**Table A8: Sensitivity of probit models for inclusion and exclusion of selected variables**

======================================================================================

Proper model No agglomeration With popul^2 No population

--------------- --------------- --------------- ---------------

Model 1 Model 2 Model 3 Model 4

--------------------------------------------------------------------------------------

(Intercept) -2.860 *** -2.856 *** -2.803 *** -2.704 ***

(0.054) (0.054) (0.055) (0.053)

locPdens.s 0.020 *** 0.023 *** 0.061 ***

(0.004) (0.003) (0.006)

locAggAgri.s -0.002 -0.005 -0.000

(0.004) (0.004) (0.004)

locAggProd.s 0.014 0.029 * 0.012

(0.013) (0.013) (0.013)

locAggConstr.s -0.101 *** -0.116 *** -0.088 ***

(0.011) (0.012) (0.011)

locAggServ.s 0.107 *** 0.103 *** 0.108 ***

(0.012) (0.012) (0.011)

locBIG.s -0.018 *** 0.006 * -0.015 ** -0.019 ***

(0.005) (0.003) (0.005) (0.005)

locHH.s -0.067 *** -0.070 *** -0.068 *** -0.062 ***

(0.013) (0.013) (0.013) (0.012)

locLQ.s 0.129 *** 0.134 *** 0.130 *** 0.111 ***

(0.003) (0.003) (0.003) (0.003)

COREfirms 0.247 *** 0.245 *** 0.218 *** 0.536 ***

(0.013) (0.013) (0.014) (0.011)

COREpopul 0.467 *** 0.462 *** 0.458 ***

(0.015) (0.015) (0.015)

dist_core_10 0.063 *** 0.060 *** 0.051 *** 0.079 ***

(0.007) (0.007) (0.007) (0.007)

dist_midsize_10 0.043 0.047 * 0.033 0.071 **

(0.022) (0.022) (0.022) (0.022)

dist_regional_10 -0.010 -0.014 -0.008 -0.006

(0.010) (0.010) (0.010) (0.010)

dist_localbig_10 -0.115 *** -0.119 *** -0.106 *** -0.123 ***

(0.006) (0.006) (0.006) (0.006)

dist_localsmall_10 0.033 *** 0.038 *** 0.036 *** 0.051 ***

(0.007) (0.007) (0.007) (0.007)

dist_core_25 0.187 *** 0.190 *** 0.180 *** 0.172 ***

(0.011) (0.011) (0.011) (0.011)

dist_midsize_25 0.117 *** 0.108 *** 0.108 *** 0.152 ***

(0.022) (0.022) (0.022) (0.022)

dist_regional_25 0.002 -0.011 -0.004 0.007

(0.007) (0.007) (0.007) (0.007)

dist_localbig_25 0.163 *** 0.167 *** 0.158 *** 0.177 ***

(0.015) (0.015) (0.015) (0.015)

dist_localsmall_25 -0.008 -0.012 -0.010 -0.003

(0.013) (0.013) (0.013) (0.013)

dist_core_50 0.151 *** 0.158 *** 0.156 *** 0.195 ***

(0.016) (0.016) (0.016) (0.016)

dist_midsize_50 0.002 0.000 0.004 -0.015

(0.020) (0.020) (0.020) (0.020)

dist_regional_50 0.072 *** 0.078 *** 0.077 *** 0.055 **

(0.018) (0.018) (0.018) (0.017)

dist_localbig_50 -0.026 -0.031 * -0.023 -0.038 **

(0.014) (0.014) (0.014) (0.014)

dist_localsmall_50 0.111 * 0.116 * 0.112 * 0.094

(0.054) (0.054) (0.054) (0.053)

locPdens.s^2 -0.029 ***

(0.003)

--------------------------------------------------------------------------------------

AIC 322492.232 322607.787 322394.741 323461.697

BIC 322799.008 322867.367 322713.316 323744.875

Log Likelihood -161220.116 -161281.893 -161170.370 -161706.848

Deviance 322440.232 322563.787 322340.741 323413.697

Num. obs. 983719 983719 983719 983719

======================================================================================

*** p < 0.001; ** p < 0.01; * p < 0.05

**Appendix 9: Classical correlation of continuous variables**

Text presented the MDS analysis which is more comprehensive than standard pairwise correlations. For comparison, Fig.A6 presents the classical Pearson correlations for continuous standardized variables. Correlation between major two variables, population density and business agglomeration, is 0.806 – this is high, but not a perfect correlation, what justifies considering those factors separately. Many pairs reveal quite low correlations as they bring diverse information. Person correlation matrix confirms the outcomes from MDS and justifies using all variables in the analysis.

**Figure A6: Pairwise Pearson correlations matrix for continuous standardized data**

|  | **locPdens** | **locAggTOT** | **locAggAgri** | **locAggProd** | **locAggConstr** | **locAggServ** | **locHH** | **locHightech** | **locBIG** | **locLQ** |
| --- | --- | --- | --- | --- | --- | --- | --- | --- | --- | --- |
| **locPdens** | 1 | 0.806 | 0.1295 | 0.78 | 0.819 | 0.809 | 0.0448 | 0.778 | 0.646 | -0.165 |
| **locAggTOTAL** | 0.806 | 1 | 0.2418 | 0.98 | 0.976 | 0.996 | 0.0711 | 0.959 | 0.881 | -0.097 |
| **locAggAgri** | 0.129 | 0.242 | 1 | 0.22 | 0.246 | 0.176 | 0.0038 | 0.122 | 0.107 | -0.074 |
| **locAggProd** | 0.781 | 0.977 | 0.215 | 1 | 0.975 | 0.973 | 0.0697 | 0.922 | 0.874 | -0.118 |
| **locAggConstr** | 0.819 | 0.976 | 0.2457 | 0.97 | 1 | 0.969 | 0.0657 | 0.919 | 0.83 | -0.127 |
| **locAggServ** | 0.809 | 0.996 | 0.1758 | 0.97 | 0.969 | 1 | 0.0723 | 0.968 | 0.889 | -0.097 |
| **locHH** | 0.045 | 0.071 | 0.0038 | 0.07 | 0.066 | 0.072 | 1 | 0.069 | 0.087 | 0.047 |
| **locHightech** | 0.778 | 0.959 | 0.1225 | 0.92 | 0.919 | 0.968 | 0.0687 | 1 | 0.844 | -0.083 |
| **locBIG** | 0.646 | 0.881 | 0.107 | 0.87 | 0.83 | 0.889 | 0.0872 | 0.844 | 1 | -0.06 |
| **locLQ** | -0.165 | -0.097 | -0.0737 | -0.12 | -0.127 | -0.097 | 0.0475 | -0.083 | -0.06 | 1 |

*All variables are continous and were standarised*

The Pearson correlation coefficients presented in a table above complement the MDS correlations presented in the text. As the relationships between the variables are partly non-linear, the Pearson correlations are only approximations and cannot be used to conclude on possible multicollinearity in probit models. MDS plot shows more robust results and evidence that each variable does not carry the same information ('bubbles' do not overlap). It should be noted that all linear estimates presented in this study must be taken with caution due to partial non-linearities and spatial heterogeneity in the data.

**References to the appendix**

Dippel, C., Ferrara, A., & Heblich, S. (2020). Causal mediation analysis in instrumental-variables regressions. *The Stata Journal*, *20*(3), 613-626.

Imai, K., Keele, L., & Tingley, D. (2010). A general approach to causal mediation analysis. *Psychological methods*, 15(4), 309.

Kopczewska, K., Churski, P., Ochojski, A., & Polko, A. (2019). SPAG: Index of spatial agglomeration. Papers in Regional Science, 98(6), 2391-2424.

Puga, D. (2010). The magnitude and causes of agglomeration economies. *Journal of Regional Science*, 50(1), 203-219.

Wyszkowska-Kuna, J. (2016). *Usługi biznesowe oparte na wiedzy. Wpływ na konkurencyjność gospodarki na przykładzie wybranych krajów Unii Europejskiej*. /Knowledge-based business services. Impact on economic competitiveness on the example of selected EU countries/ Wydawnictwo Uniwersytetu Łódzkiego. <https://bibliotekanauki.pl/books/28408782.pdf>
